# The *Y. bercovieri* Anbu crystal structure sheds light on the evolution of highly (pseudo)symmetric multimers

**DOI:** 10.1016/j.jmb.2017.11.016

**Published:** 2017-12-16

**Authors:** Anna Piasecka, Honorata Czapinska, Marie-Theres Vielberg, Roman H. Szczepanowski, Reiner Kiefersauer, Simon Reed, Michael Groll, Matthias Bochtler

**Affiliations:** 1 **Polish Academy of Sciences,** Institute of Biochemistry and Biophysics, Warsaw, Poland; 2 **School of Medicine,** Cardiff University, Cardiff, United Kingdom; 3 **International Institute of Molecular and Cell Biology,** Warsaw, Poland; 4 **Center for Integrated Protein Science at the Department Chemie,** Lehrstuhl für Biochemie, Technische Universität München, Garching, Germany; 5 **Proteros biostructures GmbH,** Martinsried, Germany; 6 **Schools of Chemistry and Biosciences,** Cardiff University, Cardiff, United Kingdom

**Keywords:** Anbu, HslV, Proteasome, Structure, Lock-washer

## Abstract

Ancestral β-subunit (Anbu) is homologous to HslV and 20S proteasomes. Based on its phylogenetic distribution and sequence clustering, Anbu has been proposed as the “ancestral” form of proteasomes. Here, we report biochemical data, small-angle X-ray scattering results, negative-stain electron microscopy micrographs and a crystal structure of the Anbu particle from *Yersinia bercovieri* (YbAnbu). All data are consistent with YbAnbu forming defined 12–14 subunit multimers that differ in shape from both HslV and 20S proteasomes. The crystal structure reveals that YbAnbu subunits form tight dimers, held together in part by the Anbu specific C-terminal helices. These dimers (“protomers”) further assemble into a low-rise left-handed staircase. The lock-washer shape of YbAnbu is consistent with the presence of defined multimers, X-ray diffraction data in solution and negative-stain electron microscopy images. The presented structure suggests a possible evolutionary pathway from helical filaments to highly symmetric or pseudosymmetric multimer structures. YbAnbu subunits have the Ntn-hydrolase fold, a putative S_1_ pocket and conserved candidate catalytic residues Thr1, Asp17 and Lys32(33). Nevertheless, we did not detect any YbAnbu peptidase or amidase activity. However, we could document orthophosphate production from ATP catalyzed by the ATP-grasp protein encoded in the *Y. bercovieri* Anbu operon.

## Introduction

Proteasomes are large macromolecular machineries that occur in all eukaryotes and carry out degradation of cytosolic and nuclear proteins that have been marked for degradation by ubiquitination [1,2]. Proteasomes are labile complexes of one or two 19S cap particles and a 20S core particle [2]. The cap is involved in specific recognition of ubiquitinated proteins, their deubiquitination, unfolding and translocation. Among other subunits, the cap contains multiple ATPases of the AAA+ type thought to be involved in substrate unfolding and translocation. The 20S proteasome is responsible for subsequent degradation of proteins. It is a 28-mer complex of at least 14 or more different subunits that together build a particle of pseudo-7-fold symmetry [3]. All 20S proteasomal subunits are paralogous and phylogenetically cluster into two groups, termed α and β. The α- and β-subunits form seven-membered rings, which assemble a particle of α_1–7_β_1–7_β_1–7_α_1–7_ architecture with a central chamber between the β-rings harboring the active sites and peripheral chambers between the α- and β-rings. The paralogy of 20S proteasomal subunits suggests evolution from simpler precursor particles. Indeed, 20S proteasomes made from just one type of α- and β-subunits have been found in archaebacteria [4,5] and some eubacteria [6–8]. A homologous protease is also present in many eubacteria, termed heat shock locus V (HslV) [9]. HslV is a 12-mer with a single central chamber containing the active sites [10,11]. The 20S proteasomes and HslV are activated by AAA+ ATPases [11–14] and have roles in protein degradation. Therefore, HslV has been regarded as the evolutionary precursor of 20S proteasomes.

This view of proteasome evolution was challenged by the bioinformatic discovery of the *An*cestral *b*eta s*u*bunit (Anbu) protein. Anbu shares distant sequence similarity with 20S proteasomes and even more distant similarity with HslV [15]. The threonine, lysine and aspartate residues that form the catalytic triad in HslV [10] and 20S proteasomes [3–5] are conserved in Anbu. Based on the scattered phylogenetic distribution of Anbu with occurrences in cyanobacteria and proteobacteria, it has been suggested that Anbu may represent an ancestral form of the 20S proteasome or HslV [15]. Alternatively, Anbu could be an evolutionary intermediate of the transition from HslV to 20S proteasomes.

Hints about the biological role of Anbu have come from contextual information and gene expression data. Anbu predominantly occurs in an operon of four genes in conserved order. The genes of the Anbu operon encode an ATP-grasp protein (with similarity to glutathione synthetase), a protein of unknown function termed alphaE (for the presence of conserved glutamates), a transglutaminase-like protein and Anbu itself [16]. Known ATP-grasp proteins, including the glutathione synthetase, catalyze amide bond formation *via* an acyl-phosphate intermediate [17]. Transglutaminases promote protein crosslinking by facilitating isopeptide bond formation at glutamine residues [18].

Bacteria contain, depending on species, either HslV or Anbu, or both particles [15]. In species that contain both proteins, Anbu and HslV are not co-regulated. HslV is upregulated upon heat shock [19], whereas this is not the case for Anbu according to microarray data [15,20]. Conversely, Anbu is highly expressed under conditions of nitrogen starvation, which do not prompt HslV expression [21].

Several hypotheses about the role of the Anbu operon have been put forward based on the available circumstantial information. The presence of two proteins that catalyze amide bond formation in the operon suggests that it may be involved in protein tagging [16]. The Anbu protein itself may have a role akin to deubiquitinating enzymes (DUBs) [16]. Alternatively, Anbu could act more like HslV or 20S proteasomes and break down substrates that have been tagged by the action of other proteins encoded in the operon [15]. Finally, there is also the possibility that Anbu may be involved in non-specific degradation of proteins in conditions of nitrogen starvation, in order to provide substrates for the transglutaminase, which generates ammonia as a side product. To our knowledge, the published hypotheses about the physiological role of the Anbu operon have not been experimentally confirmed.

## Results

### Recombinant expression and purification of Anbu proteins

Anbu genes from various bacterial species including *Yersinia bercovieri* (YbAnbu), *Pseudomonas aeruginosa* (PaAnbu), *Cytophaga hutchinsonii* (two paralogues, Ch1Anbu and Ch2Anbu) and *Ralstonia eutropha* (ReAnbu) were expressed with C-terminal histidine tags under the control of T7 promoter in the *Escherichia coli* BL21(DE3) strain. The proteins were purified by affinity chromatography on Ni-NTA agarose, followed by gel filtration on a Superose 6 column. *Hyphomicrobium sp*. Anbu (HyAnbu) was expressed and purified as described by Vielberg *et al*. Among the tested proteins, only HyAnbu and YbAnbu assembled into multi-subunit particles in our hands. In the following, we refer to Vielberg *et al*. for the characterization of HyAnbu and concentrate on YbAnbu. The protein with sequence matching the UNIPROT A0A0T9RXH3 entry (YbAnbuDB) was prone to be cleaved between S106 andG107, presumably by a protease from *E. coli*. A YbAnbu V14A/G107H variant was more stable and was therefore used in all experiments.

### YbAnbu does not form stable complexes with the other proteins encoded in the same operon

To test YbAnbu interaction with other proteins of the operon, we co-expressed all proteins in *E. coli* ER2566 strain using the pQLink system [22]. The proteins were tagged with C-terminal Myc9 tag in the case of YbAnbu, C-terminal FLAG tag in the case of transglutaminase, and either N- or C-terminal HA tag in the case of the YbATP-grasp and YbalphaE proteins. Pull-down experiments were performed using anti-Myc conjugated beads (Sigma) and YbAnbu as bait. In the pull-down fraction, we observed only Myc-tagged YbAnbu and FLAG-tagged transglutaminase. However, control experiments showed that transglutaminase bound to the beads directly. Therefore, we had to conclude that we could not detect specific interactions of the YbAnbu operon proteins.

### YbAnbu does not cleave peptidic substrates of the 20S proteasome

**Fig. 1.**
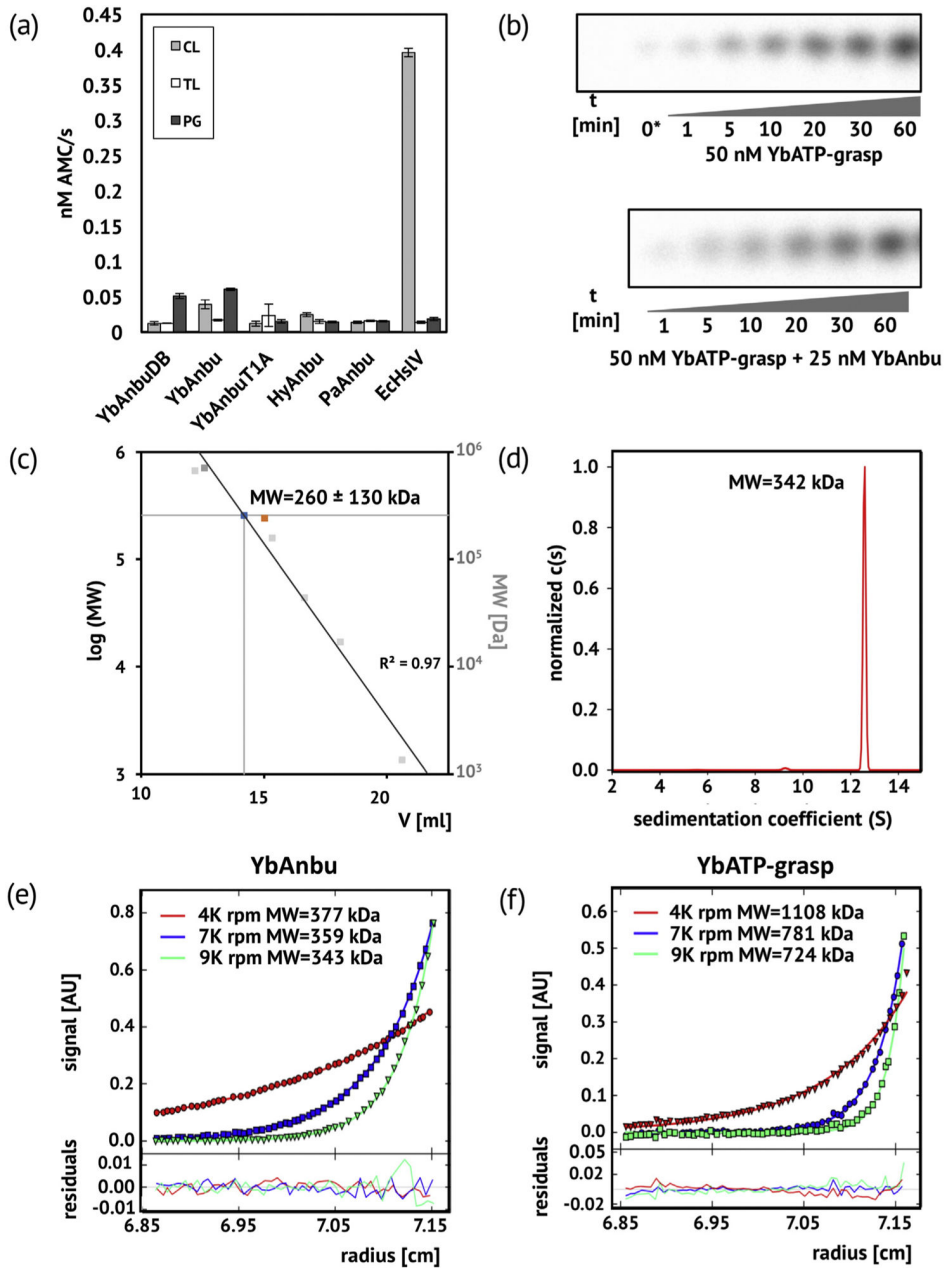
Biochemical characterization of YbAnbu operon encoded proteins. (a) Lack of Anbu activity against substrates for the 20S proteasome chymotrypsin-like (CL), trypsin-like (TL) and peptidyl-glutamyl (PG; caspase-like) activities. YbAnbuDB is YbAnbu of the exact UNIPROT sequence, YbAnbu is the Anbu otherwise used in this study (with the V14A and G107H mutations), and HyAnbu and PaAnbu are the Anbus from a *Hyphomicrobium* species and from *P. aeruginosa*. The low activity of EcHslV in the absence of EcHslU is shown for comparison. (b) Time course of YbATP-grasp catalyzed orthophosphate release from γ-P^32^-ATP in the absence (top) and presence (bottom) of YbAnbu. 0* designates the full incubation time (60 min) in the absence of enzyme. (c) Migration of YbAnbu (blue). For calibration, EcHslV (orange), Sc20S (dark gray) and BioRad gel filtration standards (light gray) were used. (d) Sedimentation coefficient of YbAnbu as determined by a sedimentation velocity experiment. (e) Radial distribution of YbAnbu in a sedimentation equilibrium centrifugation experiments at various rotational speeds. (f) Radial distribution of YbATP-grasp in sedimentation equilibrium centrifugation experiments at various rotational speeds.

Potential peptidase activity of YbAnbu was tested using standard 7-amido-4-methylcoumarin (AMC) substrates for the chymotrypsin-like (Suc-LLVY-AMC), trypsin-like (Bz-VGR-AMC) and peptidyl-glutamyl-like (Z-LLE-AMC) activities of the 20S proteasome [3]. We did the experiment for YbAnbuDB, YbAnbu, its T1A variant, HyAnbu, PaAnbu, and as a reference for *E. coli* HslV (EcHslV), which has only minimal peptidase activity in the absence of *E. coli* HslU (EcHslU) [9]. None of the Anbu molecules exhibited amidase activity against any of the tested substrates above background (Fig. 1a).

### The ATP-grasp protein in the Yb Anbu operon can release orthophosphate from ATP

ATP-grasp proteins typically catalyze a two-step reaction. In the first step, a carboxylic acid is activated to the acyl phosphate, generating ADP as a second product. Attack of an amine on the activated carboxylic acid then leads to formation of an amide bond and release of orthophosphate [17]. Differential scanning fluorimetry by the thermofluor assay [23] indicated strong destabilization of YbATP-grasp in the presence of ATP, but not ADP, suggesting that ADP and orthophosphate may be the reaction products. In order to monitor orthophosphate production, we used ATP with a radioactive ^32^P-label on the γ-phosphate and monitored the radioactive label by thin-layer chromatography followed by autoradiography. The radio-labeled ATP was incubated with the YbATP-grasp protein, either alone or in the presence of YbAnbu, EcHslV, glutamine or glutamate (urea and lysozyme were used as specificity controls). As a positive control, we also incubated the radio-labeled ATP with EcHslU, a well-characterized ATPase. The YbATP-grasp protein led to robust production of orthophosphate that was not stimulated by YbAnbu, EcHslV, glutamine or glutamate, at least under assay conditions (Suppl. Fig. 1). Production of orthophosphate could be the result of an amide bond forming reaction or ATP hydrolysis such as catalyzed by the AAA+ activators of HslV and 20S proteasome. Our experiments do not distinguish between these possibilities. For EcHslVU, it is known that EcHslV stimulates the ATPase activity of EcHslU [24]. Therefore, we performed a time course experiment to track the production of orthophosphate by YbATP-grasp in the presence and absence of YbAnbu. No difference was observed, arguing against reciprocal stimulation of YbAnbu and YbATP-grasp (Fig. 1b).

### YbAnbu and YbATP-grasp are multimers according to size exclusion chromatography and analytical ultracentrifugation

Size exclusion chromatography of YbAnbu was performed on a Superose 6 column, with *Saccharomyces cerevisiae* 20S proteasome (Sc20S) and EcHslV among the mass standards. A single elution peak was observed, at a retention time corresponding to a molecular mass of 260 ± 130 kDa (Fig. 1c). As an Anbu subunit has a theoretical mass of 28 kDa, this mass corresponds to a 9-mer but is compatible with anything from a 5-mer to a 14-mer. Although this result was repeatedly found for both YbAnbu and its T1A mutant, the 9-mer seemed implausible in the light of HslV and 20S proteasome oligomerization modes. Therefore, we carried out analytical ultracentrifugation experiments to determine the molecular mass of the YbAnbu particle. Sedimentation velocity experiments showed that YbAnbu migration was consistent with the presence of a defined multimer in solution. Fitting of the YbAnbu sedimentation profile indicated a molecular mass of 342 kDa, consistent with an Anbu 12-mer (Fig. 1d and Suppl. Fig. 2). Sedimentation velocity is influenced by molecular shape. Non-spherical or hollow particles experience more drag and sediment more slowly. Therefore measured masses can represent underestimates. In order to minimize possible shape effects, we also carried out sedimentation equilibrium experiments at three different rotational speeds (4000, 7000 and 9000 rpm). A global fit of the sedimentation profiles suggested a YbAnbu molecular mass of 368 kDa, consistent with a 14-mer particle (Fig. 1e). Similar sedimentation equilibrium distributions for YbATP-grasp indicated a relatively wide range of YbATP-grasp masses, with a global fit optimum at 780 kDa, consistent with a 14-mer in solution (Fig. 1f).

### YbAnbu crystallization and structure determination

**Fig. 2.**
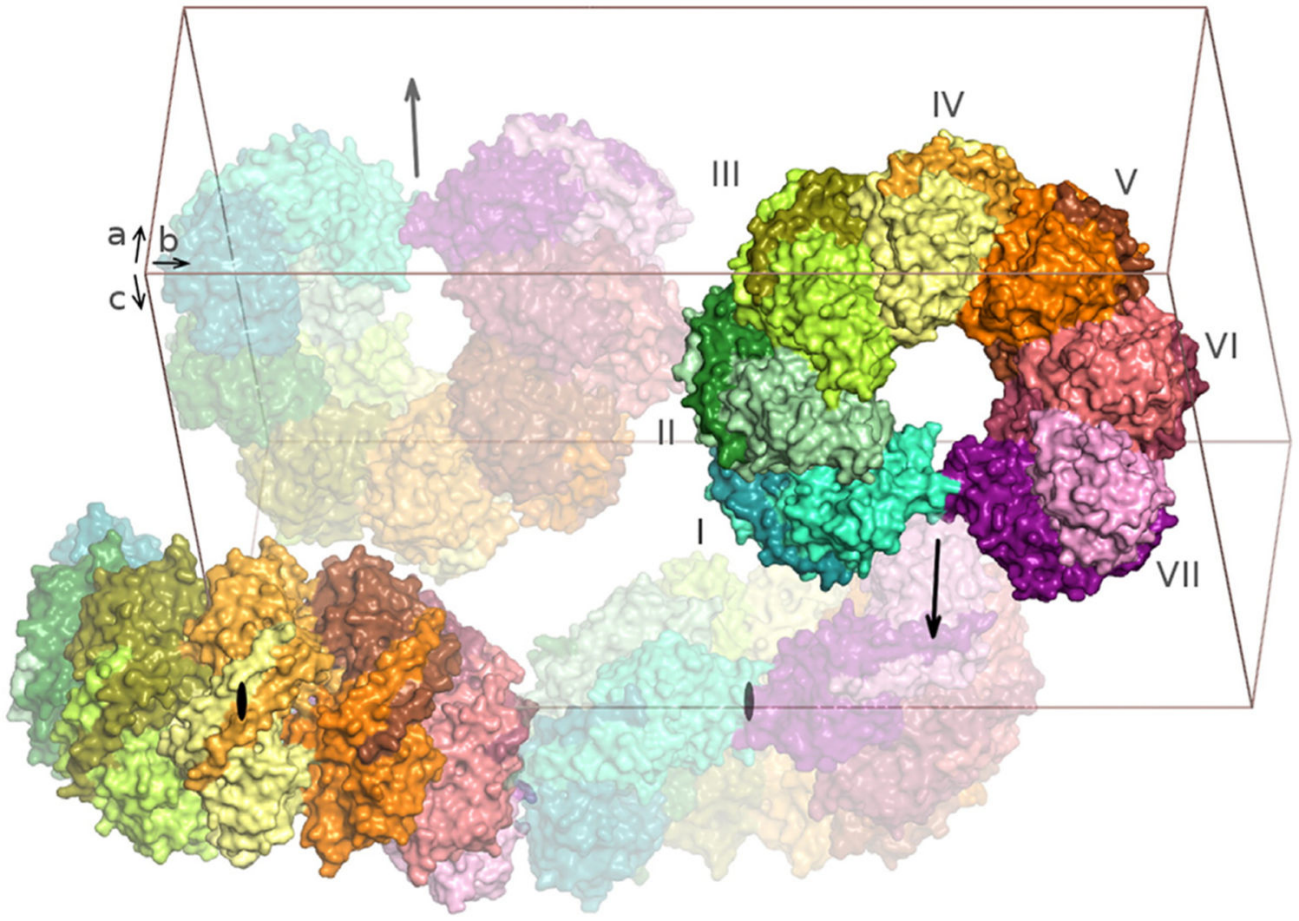
The YbAnbu multimer adopts a lock-washer shape. A view of the crystallographic unit cell is shown, with four complete YbAnbu multimers rendered in surface representation with subunit-specific colors. The Anbu basic building blocks (“protomers”) are dimers that assemble into a left-handed spiral of seven protomers (numbered from I to VII for one tetradecamer). Crystal contacts may affect the “closure” of the YbAnbu spiral. The locations of the twofold axes of the tetradecamers are indicated by arrows and ovals.

YbAnbu crystals were grown at room temperature in sitting drops. They belong to the *P2*_1_ space group (unit cell dimensions of 95.5 × 285.4 × 179.2 Å, *β* = 91.8°) and typically diffract to 3–4-Å resolution at synchrotron beamlines. Diffraction was improved by controlled dehydration at Proteros biostructures GmbH [25], leading to the collection of a dataset extending to 2.5-Å resolution at the Swiss Light Source. The structure was solved by molecular replacement using the PHASER software [26] and a subunit of HyAnbu from the *P*2_1_2_1_2_1_ crystal form as the search model (PDB ID: 5nyq). The program found 13 out of 28 Anbu molecules (corresponding to two 14-mer particles) in the asymmetric unit of the crystal. The two Anbu 14-mers were then manually completed. In order to reduce model bias, the structure was automatically rebuilt using the PHENIX [27] and ARP/wARP [28] programs and subsequently refined to the final *R*_work_ and *R*_free_ factors of 18.3 and 23.0%, respectively (Table 1). The final model coordinates and structure factors were deposited in the Protein Data Bank under accession code 5nyw (Fig. 2).

### YbAnbu monomer structure

**Fig. 3.**
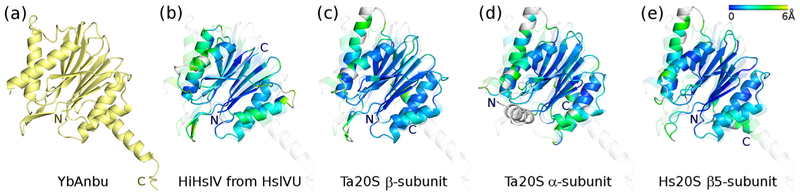
YbAnbu adopts the β-sandwich fold of HslV and 20S proteasomes, and differences are most pronounced at the C-terminus. The figure shows a single protomer of YbAnbu in yellow and superpositions of HiHslV (PDB ID: 1g3i [13], chosen because it shows the active form taken from the complex with HiHslU) and of various 20S subunits (PDB IDs: 1pma [4] and 4r67 [30]) on YbAnbu (faint). In the superposition panels, the structures are color-ramped from blue to green according to the rmsd between corresponding residues (light gray color is used for residues with no correspondence).

YbAnbu exhibits the β-sandwich fold with a characteristic four-layered architecture. The two central layers are β-sheets stacked on top of each other and wrapped by two layers of α-helices. Comparisons performed with the help of the PDBeFold server [29] confirm that YbAnbu is structurally most similar to 20S proteasome β-subunits, and slightly less similar to HslV (*Z*-scores for the best matching subunits in the range of 11 and 8). The proteins differ most in the region of the C-terminus [15]. The 20S β-subunits have an additional β-strand and α-helix compared to HslV, which is thought to affect the subunit packing and assembly into hexameric or heptameric rings [10]. In Anbu, the C-terminal helix is much longer than in 20S proteasomes and appears to play a major role in multimer assembly, as it mediates intersubunit contacts in the particle (Fig. 3).

### Putative active site of YbAnbu

**Fig. 4.**
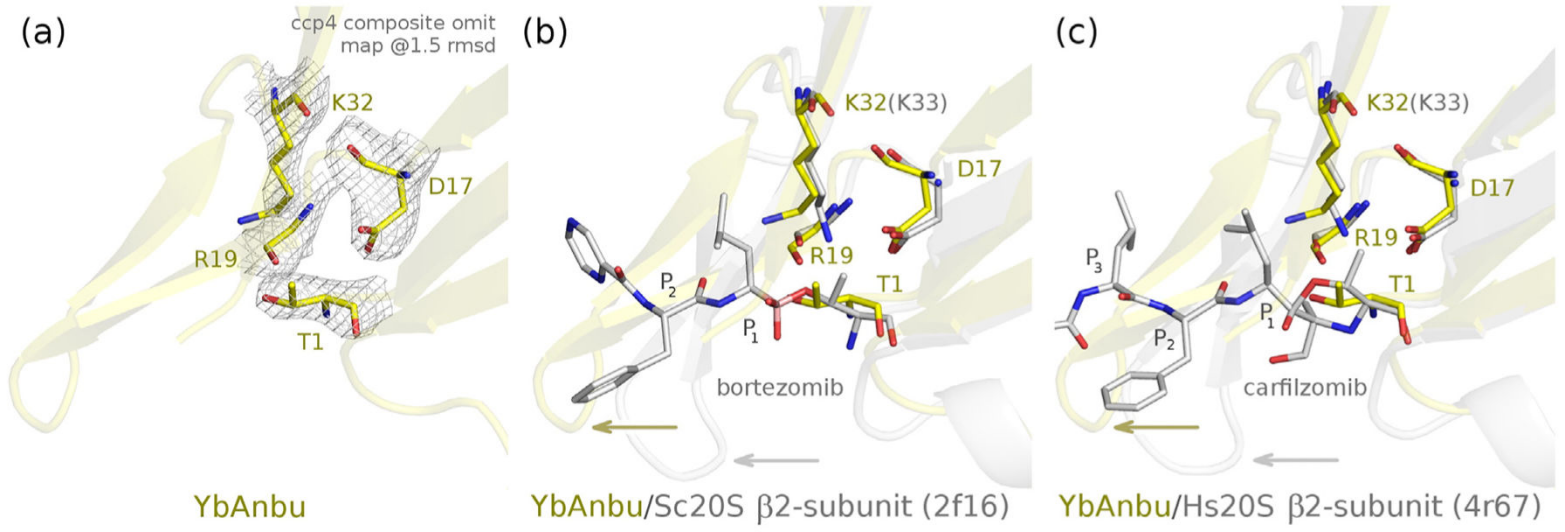
Proteasome active site residues are conserved in YbAnbu and adopt similar conformations, a hairpin of YbAnbu clashes with the P_2_ and P_3_ substrate/inhibitor residues in the predicted binding mode. (a) Region of YbAnbu in the vicinity of the N-terminal threonine in ribbon representation, with key residues shown in stick representation in a composite omit map contoured at 1.5 rmsd. (b, c) Superpositions of YbAnbu (yellow) on *S. cerevisiae* (Sc20S) and Homo sapiens 20S (Hs20S) proteasome β2-subunits bound to bortezomib and carfilzomib (gray, PDB IDs: 2f16 [32] and 4r67 [30]). The side chain of R19 is omitted for clarity. Note the clashes of the P_2_ and P_3_ residues of bortezomib and carfilzomib with a hairpin of YbAnbu, but not the equivalent hairpins in Sc20S and Hs20S (yellow and gray arrows).

YbAnbu (as other Anbus) retains key features of 20S proteasomes and HslV associated with their catalytic activities. 20S proteasomes and HslV are Ntn-hydrolases that mature by cleavage of a proregion or initiator methionine to expose an N-terminal threonine residue that acts as a nucleophile and general base [5,31]. In addition, the active sites contain an aspartate and a lysine residue that are also required for activity. All three residues are conserved in YbAnbu (T1, D17 and K32). We have confirmed by mass spectrometry that the YbAnbu T1 is exposed by cleavage of the initiator methionine residue. A comparison of crystal structures shows that all three putative catalytic residues adopt similar conformations in YbAnbu and in active proteasomes (Fig. 4).

However, the crystal structures also indicate some differences. In the YbAnbu structure, the electron density for most of the lysine residue is clear, but the Nε-amino group is poorly defined. Due to steric constraints, the amino group has been tentatively modeled away from the aspartate and hydrogen bonded to other residues in the vicinity with some variation between subunits (Suppl. Fig. 3A). However, we note that the lysine residue also adopts varying positions in proteasome structures without substrates or substrate analogues, and therefore, the slightly different conformation may not be the cause for the lack of activity (Suppl. Fig. 3). We also note that a mild deformation of a hairpin (residues 17–31) suffices to achieve good superposition between YbAnbu and other proteasome structures.

### Potential substrate binding site of YbAnbu

As the putative catalytic residues of YbAnbu are approximately in the expected locations, we looked at the substrate binding sites for clues to explain the observed lack of activity toward proteasomal substrates. Both 20S proteasomes and HslV interact predominantly with the non-primed side of substrates (the region upstream of the scissile bond). Peptidic substrates and inhibitors are bound in the β-strand conformation. They extend the β1-strand that carries the active site threonine, and engage in hydrogen bonds with another β-strand of the enzyme [33]. The region involved in substrate binding interactions is present in YbAnbu (residues 19–23). Therefore, we expect that potential peptidic substrates of YbAnbu may be bound analogously.

### YbAnbu has a counterpart for the S_1_ pockets of HslV and 20S proteasomes

**Fig. 5.**
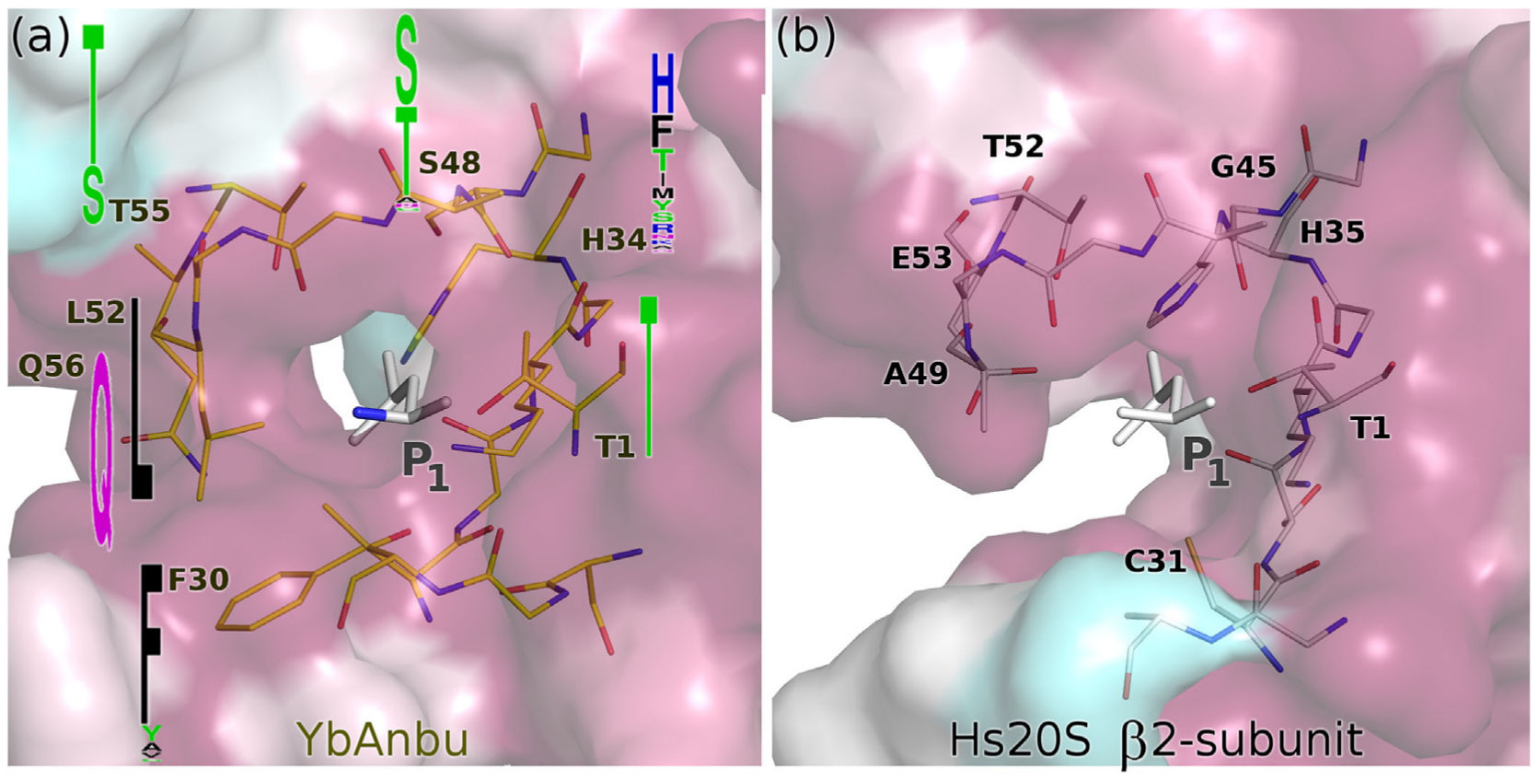
The YbAnbu S_1_ pocket resembles the proteasomal β2-subunit pocket, but is narrower. (a) Putative S_1_ pocket of YbAnbu with P_1_ residue mapped from the co-crystal structure of Hs20S with carfilzomib (PDB ID: 4r67 [30]) based on global superposition of subunits. Key residues are shown in stick representation, and their conservation among Anbu proteins from different species is indicated by sequence logos in frequency mode. (b) S_1_ pocket of Hs20S β2 subunit with bound P_1_ residue (the rest of the carfilzomib is omitted for clarity). The protein surfaces have been colored according to sequence conservation from blue (low) to red (high). For Anbu, the underlying sequence logo is presented in Suppl. Fig. 5.

For the well-characterized proteasomes, substrate specificity is primarily determined by the P_1_ residue (the residue immediately upstream of the scissile amide bond) [33,34]. The (putative) S1 pocket is smaller in YbAnbu than in HslV and 20S proteasome subunits due to altered amino acid composition, and other conformations of the β-hairpin region (residues 17–31) and (putative) catalytic lysine residue (K32). The YbAnbu pocket is deep and has the aliphatic region of lysine (K32), serine (S48) and threonine (T55) as side walls and a histidine (H34) and leucine (L52) at the bottom. Serine 48 is structurally equivalent to the residue 45 in 20S proteasomes that is thought to contribute to subunit specificity [3]. A histidine is located deep at the bottom of the putative YbAnbu S_1_ pocket. This may suggest a caspase-like activity, or by analogy/homology to Sc20S β2 (which also has a histidine in the equivalent position [3]), a trypsin-like specificity. However, the histidine is not conserved in YbAnbu proteins from other species and is therefore unlikely to be the key specificity determinant (Fig. 5 and Suppl. Figs. 4 and 5).

Due to alternating side-chain directions in β-strands, the P_2_ residue of substrates, peptidic substrate mimics or inhibitors points away from the protein into the cavity of proteasomes and is therefore not expected to play a major role in substrate selection in any proteasome-like particle. The side chain of the P_3_ residue, however, is directed toward the protein. In YbAnbu, the hairpin connecting the β2 and β3 strands (residues 17–31) would collide with the P_2_ and P_3_ residues in “canonical” position. However, we note that the same hairpin is also present in 20S proteasomes, except that it adopts a slightly different conformation that leaves space for substrates (Suppl. Fig. 6). In 20S proteasomes, clashes are avoided even when the inhibitors are modeled into structures that were determined in the absence of ligands, possibly suggesting a genuine difference between YbAnbu and proteasomes. However, the clashing hairpin adopts 20S-like conformation or is disordered in the other Anbu structures (Suppl. Fig. 7). Therefore, it is difficult at this point to assess the importance of the observed clashes.

### YbAnbu oligomerization

The basic building block of the YbAnbu particle appears to be a protomer built from two subunits tightly bound to each other, in part by interactions between the Anbu specific C-terminal helices. The YbAnbu protomers are then further assembled into a left-handed helical staircase. Due to the low rise of the helical arrangement, the filament of protomers cannot be extended indefinitely. Instead, 7 protomers or 14 subunits assemble into a twofold symmetric lock-washer shaped particle (Fig. 2).

**Fig. 6.**
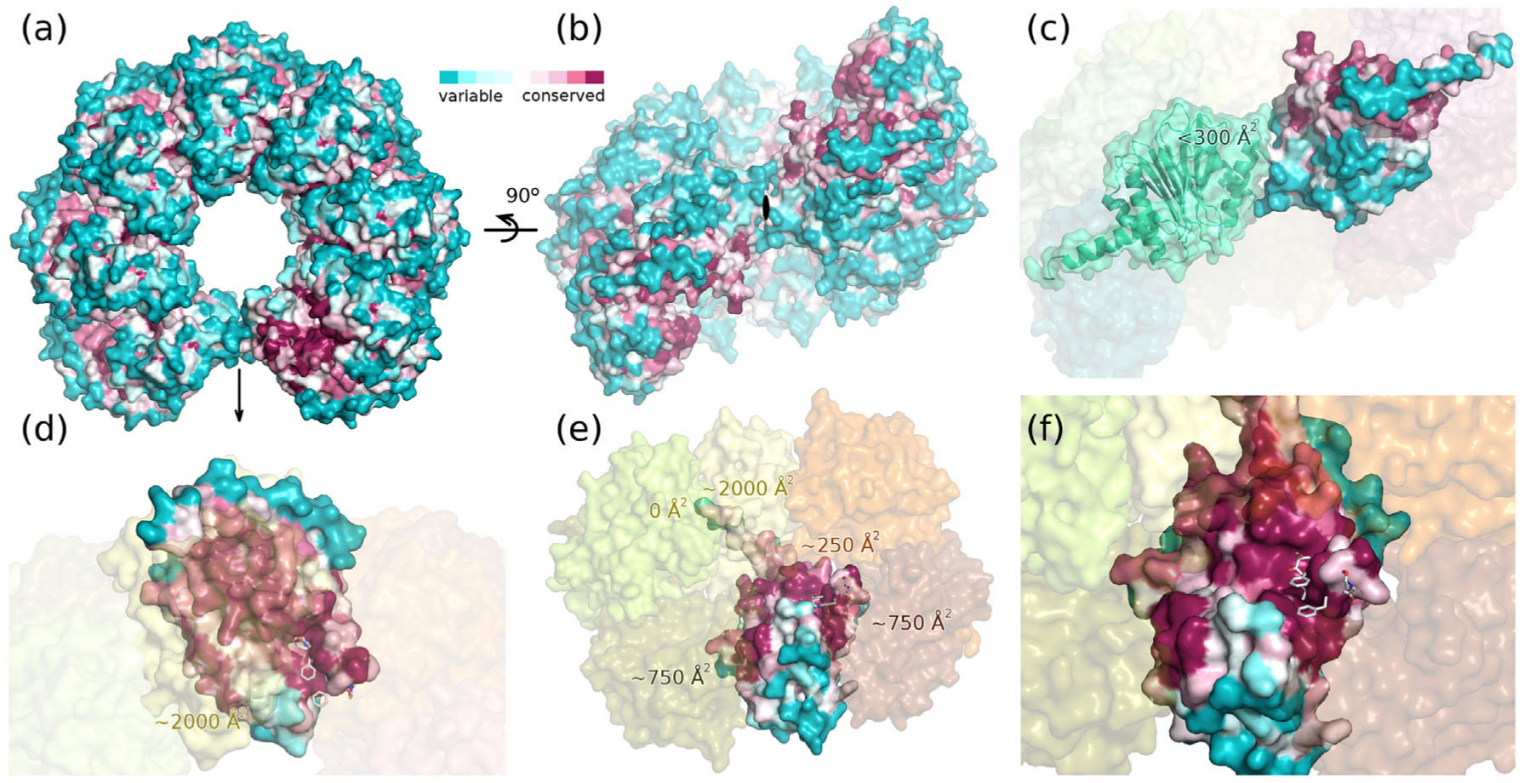
YbAnbu shape and surface conservation. (a) Top view of the YbAnbu surface colored according to sequence conservation as in Fig. 5. The bottom view is identical due to an internal twofold axis running vertically (indicated by an arrow). Note the conservation of the sites that would be docking surfaces for another protomer if it did not clash with the other end of the lock-washer. (b) Side view of the YbAnbu surface colored analogously. YbAnbu subunits are arranged in a left-handed spiral of low rise. (c) Contact of the subunits at the ends of the lock-washer. The interaction surfaces are not conserved, and many contacts among the two subunits are not favorable. (d) Magnified view of panel a with only a single protomer highlighted and the surface of its top subunit shown in transparent light yellow to show its footprint. The surface of the bottom subunit colored as in panel a and all other subunits are indicated with faint transparent surfaces. (e) Magnified view of panel b with subunits near the closure of the spiral removed to show subunit interactions and conservation of the inside of the lock-washer. (f) Magnified view in a similar orientation to that in panel e to show conservation around the T1 residue. The carfilzomib molecule has been mapped based on the Hs20S structure to indicate potential substrate binding mode and is not part of the crystal structure.

Among Anbu particles, the outer surface and the ring closing interface are not conserved. (Fig. 6a, b). The ring closing interface area is not extensive (<300 Å^2^) and built by contacts that do not look favorable (Fig. 6c). In contrast, both the intra-protomer and the inter-protomer interfaces are extensive (2000 and 1000 Å^2^, respectively) and highly conserved (Fig. 6d, e). The putative active site groove of the Anbu particle is solvent exposed and conserved arguing for an enzymatic activity of the protein (Fig. 6f).

**Fig. 7.**
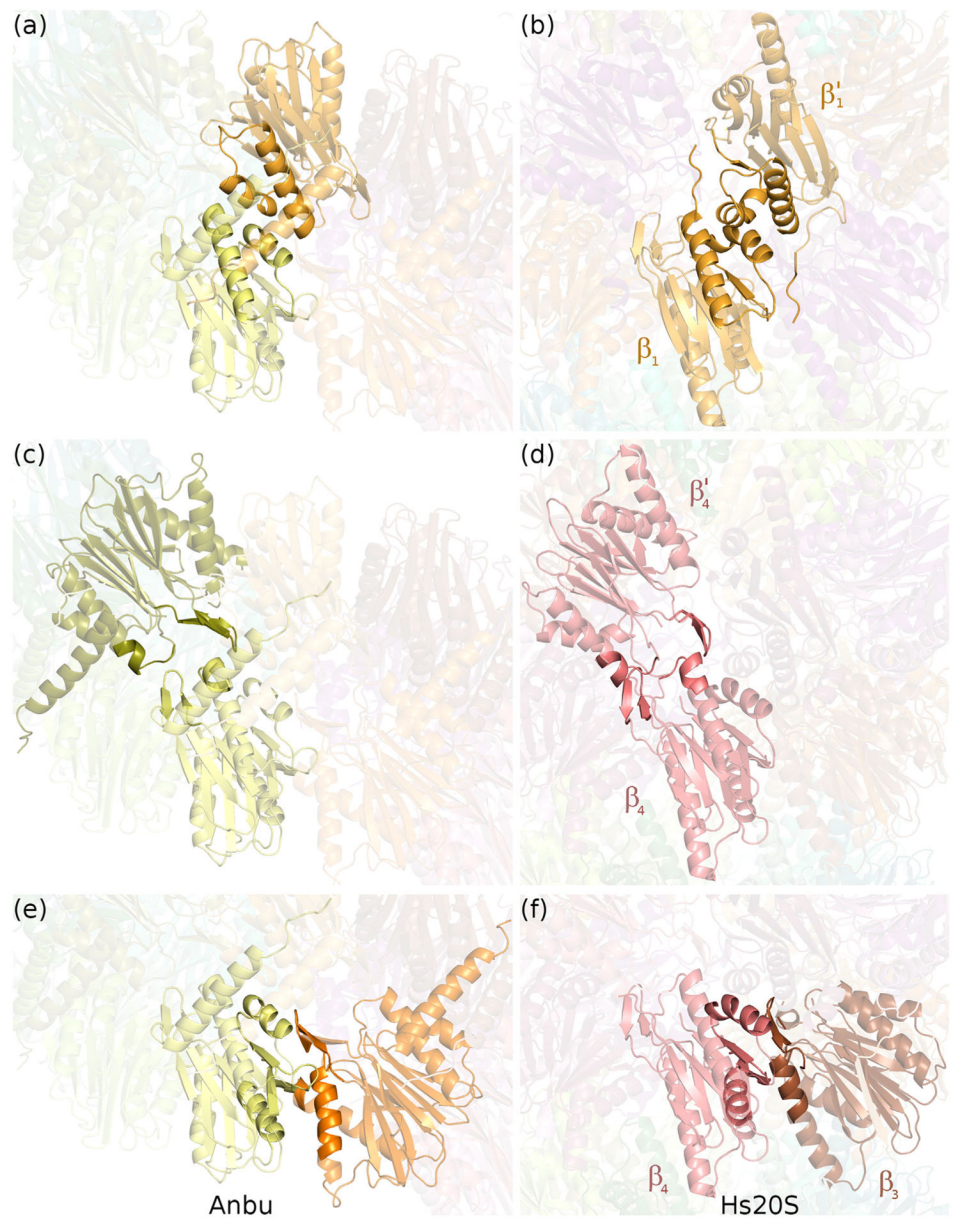
Similar local interfaces between YbAnbu and 20S proteasome β-subunits. Secondary structures responsible for intra-protomer contacts (a, b) and for interactions with the neighboring protomers (c, d) across rings and (e, f) within the same ring are presented in the cartoon mode. The contacts of YbAnbu protomer are shown on the left and their counterparts in 20S proteasomes on the right. YbAnbu is built of one type of subunits, and thus, all but the lock-washer closing contacts are very similar. In the case of the proteasome, the twofold symmetry-related subunits have been chosen in panel b (β1 and β1′ subunits of caspase-like specificity) and d (β4 and β4′ subunits).

A comparison of the YbAnbu particle with 20S proteasome β-rings shows that small differences in overall shape result from small local changes. Protomers are very similar (Fig. 7a, b), and even inter-protomer contacts (both diagonal and lateral) do not differ much (Fig. 7c–f). Small differences apparently suffice to control assembly into either rings or spirals.

### The lock-washer shape of YbAnbu is consistent with small-angle X-ray scattering and negative-stain electron microscopy data

**Fig. 8.**
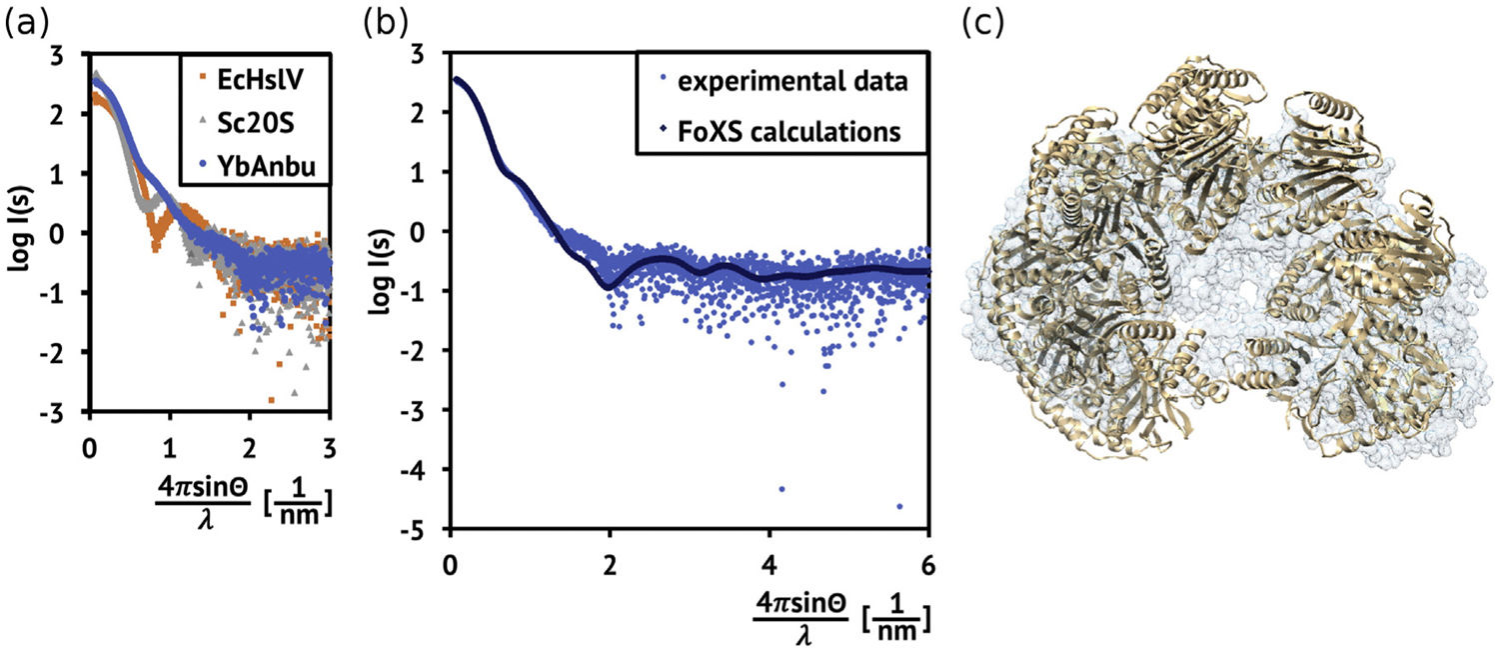
Shape of YbAnbu in solution. (a) Deflection-dependent decrease of scattering intensity from YbAnbu (blue), EcHslV (orange) and Sc20S (gray). (b) Comparison of measured and predicted SAXS data for YbAnbu. Calculations were done using the FoXS sever [36]. (c) Fitting of the YbAnbu crystal structure into the envelope determined by *ab initio* interpretation of the SAXS data using GASBOR [37].

Small-angle X-ray scattering (SAXS) data in solution were obtained for YbAnbu and as internal controls for EcHslV or Sc20S. CRYSOL [35] and FoXS [36] servers indicated a good match between the measured solution diffraction data and crystal structure-based predictions [3,10], as judged from the *X* values (1.16 for EcHslV and 1.61 for Sc20S; Suppl. Fig. 8). YbAnbu behaved unlike EcHslV or Sc20S in solution scattering experiments. YbAnbu scattering did not show any minima in the region of low photon momentum transfers (*s* < 1.0/nm, *s* = 4πsin(θ)/λ, where 2π is the X-ray deflection angle and λ is the X-ray wavelength). At *s* > 1.0/nm, the YbAnbu scattering was notably less dependent on photon momentum transfer (Fig. 8a). The agreement between measured and calculated SAXS data for YbAnbu was fair (*X* = 2.86) (Fig. 8b), but not as good as for EcHslV or Sc20S. Reassuringly, the lock-washer shape of YbAnbu was also reproduced by *ab initio* interpretation of the SAXS data using the GASBOR server [37]. The crystal structure and *ab initio*-determined shape agreed well, according to superposition by the SUBCOMP program [38] (Fig. 8c).

**Fig. 9.**
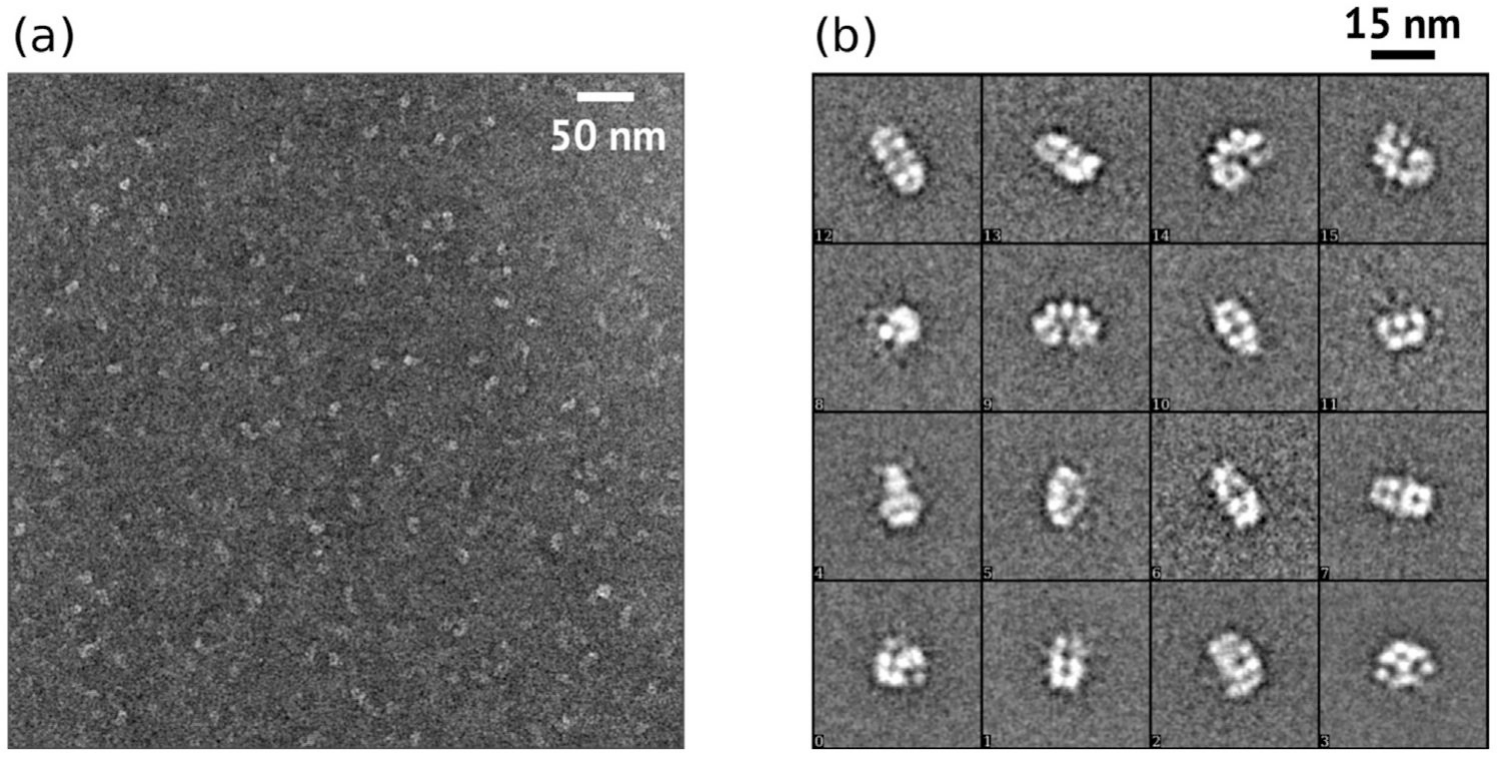
YbAnbu appears heterogeneous in negative-stain EM micrographs, and lock-washer-shaped particles are frequent. (a) Selected region of a negative stain grid. (b) Reference-free 2D class averages of YbAnbu particles. Many views can be interpreted as “top” and “side” views of a lock-washer-shaped particle.

In negative stain electron micrographs, YbAnbu appeared to be rather heterogeneous. Among the class averages, we noted a lock-washer-like particle consistent with the shape determined by X-ray crystallography and by *ab initio* interpretation of the SAXS data. The more rectangular shapes may represent views from the side (Fig. 9). Neither HslV nor 20S proteasome-shaped particles were observed in the YbAnbu micrographs.

## Discussion

### Potential Anbu activities

Conservation of all active site residues of 20S proteasomes and HslV in YbAnbu suggests a possible peptidase or amidase activity. Occlusion of the S_2_ and S_3_ binding sites may be an explanation for the lack of activity of YbAnbu against peptidic substrates of the 20S proteasomes, but it is not observed in all Anbu structures. Whether shorter chromogenic peptides could be substrates for YbAnbu has not yet been tested. We note that Anbu need not necessarily act as a peptidase or isopeptidase. The β-sandwich fold and N-terminal nucleophile are not only shared between HslV and 20S proteasomes, but also the hallmarks of a wider class of so called Ntn-hydrolases (although the nucleophile is not necessarily a threonine) [31]. Some Ntn-hydrolases cleave amide bonds that are not peptide bonds. Examples include penicillin acylase [39] or aspartylglucosaminidase (*N*(4)-(beta-*N*-acetylglucosaminyl)-L-asparaginase), an aminohydrolase involved in the catabolism of N-linked oligosaccharides of glycoproteins [40]. As the Anbu operon encodes also a transglutaminase, it is remarkable that some Ntn-hydrolases act as glutamine amidotransferases that catalyze hydrolysis of the side-chain amide of glutamine and subsequent transfer of the amino group to various acceptors. Examples of such Ntn-hydrolases include glucosamine-6-phosphate synthase [41] and glutamine 5-phosphoribosyl-1-pyrophosphate amidotransferase [42]. However, the amidotransferases have an extra subunit in addition to the Ntn-hydrolase domain that is absent in Anbu. Moreover, among the Ntn-hydrolases, Anbu resembles most closely the 20S proteasome β-subunits, and thus, a peptidase or amidase activity is still most likely.

### Potential Anbu activation

The lack of YbAnbu peptidase activity is reminiscent of the very poor peptidase activity of HslV in the absence of HslU [9]. Structural studies have shown that activation of HslV peptidase activity by HslU involves insertion of the C-terminal tails of HslU subunits into clefts between HslV subunits. This interaction brings about subtle conformational changes that stimulate HslV peptidase and HslU ATPase activities [13,24]. A AaA+ ATPase is not encoded in the YbAnbu operon, and YbAnbu activators have not yet been identified. Nevertheless, we wondered whether YbAnbu might have similar groves as HslV that could potentially accommodate an activator protein. Therefore, we mapped the HslVU complex on YbAnbu, aiming for optimal superposition of one YbAnbu on one HslV subunit. The superposition shows that HslU C-terminal tail could indeed be inserted in the groove between YbAnbu subunits (Suppl. Fig. 9). We also noted a patch of conserved hydrophobic and charged residues reminiscent of the C-terminus of HslU in the C-terminal region of the ATP-grasp protein, but the latter extends beyond the analogous region. Moreover, YbAnbu did not stimulate orthophosphate production from ATP by the ATP-grasp protein. Therefore, the current data do not support YbAnbu activation by ATP-grasp akin to HslV activation by HslU [9], but does not exclude other activatory mechanisms. EcHslVU activity is several-fold enhanced by the presence of potassium ions [43], which are coordinated by main-chain carbonyl oxygen atoms in the vicinity of the active site [44]. The cation binding site is conserved in 20S proteasomes, where it tends to bind Mg^2+^ ions [3] and in YbAnbu. In the YbAnbu structure, we can see a solvent molecule bound in most subunits, but the resolution is insufficient to distinguish between water and metal cations (Suppl. Fig. 10).

### Comparison of the YbAnbu structure with other Anbu structures

While we were in the process of preparing this manuscript, a publication by Fuchs *et al*. [45] appeared online describing the structure of *P. aeruginosa* Anbu (PaAnbu) and of a designed Anbu with amino acid sequence based on the consensus of available Anbu amino acid sequences (ConsAnbu). Together with the HyAnbu structures reported in the accompanying publication by Vielberg *et al*. and the structure reported in this work, many Anbu structures are now available.

All Anbu crystal forms contain either two subunit protomers or left-handed helical filaments of such protomers. Spiral staircases that could be continued indefinitely are seen in the crystal structures of PaAnbu and ConsAnbu [45] and in the *C2* and *P*2_1_2_1_2_1_ crystal forms of HyAnbu (accompanying manuscript by Vielberg *et al*.) (PDB IDs: 5lox, 5loy, 5nyj and 5nyg, respectively). Such filaments have also been observed in electron microscopy (EM) micrographs by Fuchs *et al*. [45] after artificially stabilizing the arrangement seen in the crystals by engineered disulfide bonds. However, spiral filaments have not been detected in solution for naturally occurring enzymes. We suspect that crystallization favors helical assemblies, for “contact number” reasons similar to those favoring screw over non-screw rotations in protein crystals [46]. Non-physiological spiral formation in crystals by subunits that “should” assemble into planar structures has precedence, for example, in the case of ClpA [47].

The YbAnbu structure reported in this work differs from all other helical assemblies observed for Anbu by its much smaller rise. As in a lock-washer, the ends of the helical filament are in touch, naturally limiting polymerization. The 14-mer structure is compatible with molecular mass determinations that all suggest a defined multimer. Moreover, it is also supported by SAXS and EM data. We note that the lock-washer shape of YbAnbu agrees remarkably well with the shape that Fuchs *et al*. have deduced for PaAnbu by adjusting the orientation of PaAnbu subunits to fit the SAXS data for this particle [45]. In summary, it appears that Anbu particles form helical assemblies that exhibit substantial variability in shear. We suspect that small helical rises such as seen in the YbAnbu crystal structure predominate in solution. At present, it remains unclear whether susceptibility of Anbu multimers to shear has biological relevance.

### A lock-washer structure is an intermediate on the way toward highly (pseudo)symmetric multimers

YbAnbu, HslV and 20S proteasome β-rings are locally very similar. The protomer building block of YbAnbu is also present in HslV and 20S proteasomes. Local contacts, both within rings and across rings, are conserved. The hierarchy of these contacts is surprising, though. The tight association of subunits in the Anbu protomer suggests that protomers assemble first and then arrange themselves into a helical superstructure. In contrast, inter-ring contacts are established last in the assembly pathway of most [48,49], but perhaps not all 20S proteasomes [50].

Although locally YbAnbu, HslV and 20S proteasome β-rings make very similar contacts, the overall shapes of the particles are rather different. YbAnbu ring dimensions are slightly larger than for HslV and 20S proteasomes, due to the larger number of subunits per ring relative to HslV and to the “open” form of the ring. The smaller ring radius of HslV compared to 20S proteasomes has been attributed to the less bulky C-terminus [10]. The larger ring radius of YbAnbu compared to HslV and 20S proteasomes may represent the same effect. Moreover, YbAnbu has a helical twist and lacks the high (pseudo) point symmetry of 20S proteasomes and HslV. Thus, only the twofold symmetry is shared between the two types of particles (Suppl. Fig. 11).

**Fig. 10.**
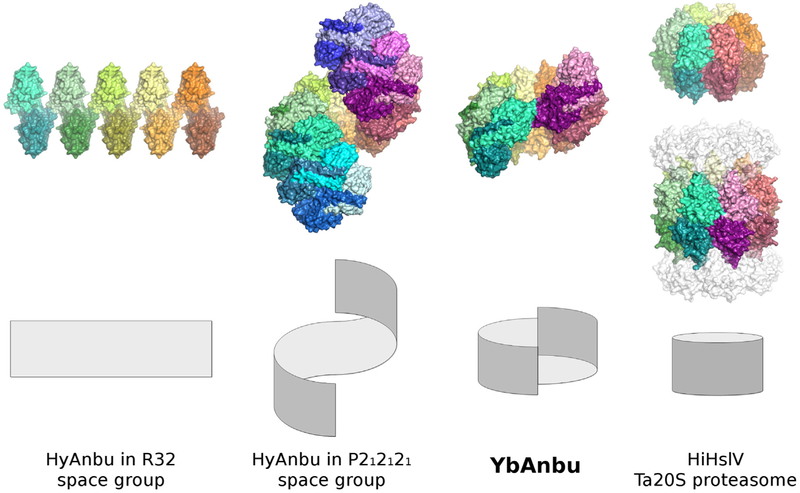
A helically twisted lock-washer could be an intermediate “on the way” to a ring. Two-layered sheets of subunits may be bent and twisted to form spirals. A large rise should lead to polymerization, and a smaller rise should lead to lock-washer-shaped structures. Ring structures are formed in the absence of rise.

The most prominent difference between YbAnbu on the one hand and HslV and 20S proteasomes is the shear of the two rings of subunits and the loose association between ends. The structure of Anbu as a curved “sheet” wrapped back onto itself is nicely compatible with the evolutionary scenario that depicts Anbu as a relic that retains features of an ancestral proteasome. According to this point of view, both sixfold symmetric HslV and sevenfold symmetric or pseudo-symmetric 20S proteasomes could have evolved from a multimer with imperfect ring closure. Better stability and improved sequestration of an internal cavity from the outside world could have been among the benefits that might have driven such a change (Fig. 10).

## Materials and Methods

### Anbu cloning, expression and purification

The YbAnbu gene was amplified from *Y. bercovieri* (DSM 18528; ATCC 43970) using PCR with primers introducing NdeI and XhoI restriction sites and cloned *via* these enzymes into pET20b vector (Novagen) encoding C-terminal His-tag. Protein overexpression was performed in the *E. coli* BL21(DE3) strain. Cells were grown in LB medium supplemented with ampicillin at 28 °C o/n. They did not require IPTG induction probably due to promoter “leakage.” Immediately after harvesting, cells were re-suspended in buffer A [50 mM Tris–HCl (pH 7.5), 200 mM NaCl] and disrupted by sonication or using French press. The supernatant was clarified by centrifugation at 134,000*g*, supplemented with 100 mM imidazole and applied on Ni-NTA column (Qiagen). After an extensive wash of the column with buffer A containing 100 mM imidazole, the protein was eluted with buffer A with 500 mM imidazole. Immediately after elution, 1 mM EDTA (pH 8.0) was added to the protein solution. YbAnbu protein was concentrated using VivaSpin 100,000 MWCO concentrators and applied on a Superose 6 column equilibrated with buffer B [20 mM Tris–HCl (pH 7.5), 15 mM NaCl, 1 mM EDTA]. YbAnbu eluted as a multimer.

SDS-PAGE analysis revealed that wild-type YbAnbu was partially inhomogeneous due to proteolytic digestion. The cleavage site was identified by mass spectrometry (between S106 and G107). Site-directed mutagenesis was used to generate YbAnbu variants with randomly altered residues immediately upstream and downstream of the scissile amide bond. The YbAnbu G107H variant (with the additionally introduced V14A exchange) was less susceptible to proteolysis than the wild-type enzyme and was therefore chosen for further studies. All work presented in this publication was performed using this variant. The T1A variant of Anbu with alanine instead of the N-terminal threonine was prepared by the QuikChange mutagenesis protocol. The T1A YbAnbu variant was expressed and purified as described above.

Cloning, expression and purification of PaAnbu, Ch1Anbu, Ch2Anbu and ReAnbu were similar to YbAnbu. HyAnbu was expressed and purified as described in the accompanying Vielberg *et al*. publication.

### Expression and co-immunoprecipitation of the proteins encoded by the Anbu operon

For co-immunoprecipitation studies we co-expressed proteins of the YbAnbu operon. For co-expression in *E. coli*, we used the pQLink vectors containing genes of the operon with various tags. YbAnbu did not express from the pQLink vectors in our hands. Therefore, an open reading frame for C-terminally Myc3-tagged YbAnbu was placed into pET20b and then cloned into the pQLinkN vector together with the T7 promoter using BsaBI and BlpI restriction sites.

Other genes of the YbAnbu operon were amplified by PCR from the native source (DSM 18528; ATCC 43970) and cloned into modified pQLink vectors harboring HA- and FLAG-tags (GGATCCgaattcACTAGTggtTACCCATACGATGTTCCAGATTACGCTggtAGATCTatatgCCTAGG for HA-tag and GGATCCgaattcACTAGTggtGATTACAAGGATCACGACGGCGACTACAA GGACCATGACATTGATTACAAGGACGATGACGACAAAggtAGATCTcatatgCCTAGG for FLAG-tag cloned with *Bam*HI and *Avr*II into pQLinkN). Depending on the chosen restriction sites (BamHI/BglII, XhoI/AvrII), the tag could be placed on either N- or C-terminus of the protein. In this way, we prepared N- and C-terminally HA-tagged YbalphaE and YbATP-grasp, as well as Ybtransglutaminase with C-terminal FLAG-tag.

The plasmid for co-expression of all four open reading frames (from independent promoters) was assembled according to the pQLink protocol. Expression was performed in ER2566 cells. After reaching OD600 = 0.5, cells were first kept for 30 min at 42 °C, then cooled to 37 °C, induced with IPTG (final concentration 1 mM), and cultured for another 2 h. Cells were re-suspended in the IP buffer (Anti-c-Myc Immunoprecipitation Kit, Sigma) and mildly sonicated in a bath sonicator for 30 min. The co-immunoprecipitation was performed according to the manufacturer's recommendations. Proteins were visualized by Western blots using tag-specific antibodies.

### YbAnbu activity assay

YbAnbu activity tests were performed using fluorogenic substrates Suc-LLVY-AMC (chymotrypsin-like activity), Bz-VGR-AMC (trypsin-like activity) and Z-LLE-MCA (caspase-like activity). Reactions were carried out in buffer C [50 mM Tris (pH 7.5), 300 mM NaCl, 5 mM MgCl_2_] with 10 μM substrate and 1 μM protein on a 96-well plate at 37 °C for 60 min. The fluorescence of the free AMC was measured with an excitation wavelength of 355 nm and an emission wavelength of 460 nm. Errors in Fig. 1A are standard errors for three experiments and do not include the error of the slope of the calibration curve for AMC concentration, which we expect to be around or below 10%.

### ATP-grasp cloning, expression and purification

The YbATP-grasp gene was amplified by PCR from native source and cloned into pQlinkH vector using BamHI and AvrII restriction sites. Plasmid harboring N-terminally His-tagged YbATP-grasp was then transformed into ER2566 cells. Cells at OD_600_ of 0.3 to 0.5 were supplemented with ethanol to 2% final concentration and induced 30 min later with 0.5 mM IPTG. Protein expression was done at 37 °C overnight. After harvesting, cells were stored at −20 °C until further use.

YbATP-grasp protein was purified similarly to YbAnbu on Ni-NTA. The only difference was in the loading and washing steps, where the solutions were supplemented with 20 mM imidazole. Eluted protein was diluted with a threefold excess of buffer B and applied to a MonoQ column. After an extensive wash with buffer B, the protein was eluted with a gradient from 20 mM to 1 M NaCl. YbATP-grasp that eluted at ~350 mM NaCl was then concentrated and applied on the Superose 6 column equilibrated with buffer D [50 mM Tris (pH 7.5), 300 NaCl, 1 mM EDTA].

### YbATP-grasp activity assay

YbATP-grasp activity on ATP was tested in buffer E [50 mM Tris (pH 7.5), 150 mM KCl, 5 mM MgCl_2_, 1 mM ATP]. Purified YbATP-grasp (0.7 μg) alone or mixed with 0.4 μg YbAnbu was incubated with 0.4 μl γ-P^32^-ATP in a final volume of 40 μl for various periods of time at RT. At various times, 5 μl aliquots of the reaction were withdrawn, and the reaction in these aliquots was stopped by the addition of 5 μl 0.7 M LiCoOH and 1 M HCOOH. The reaction products were then resolved on TEI Cellulose F plates in running buffer (0.35 M LiCOOH, 0.5 M HCOOH) and visualized by autoradiography.

### Gel filtration analysis

Gel filtration was performed on Superose 6 30/100 column (0.5 ml/min flow rate) equilibrated with buffer D. The column was calibrated using BioRad Gel Filtration Standards (thyroglobulin 670 kDa, bovine gamma-globulin 158 kDa, chicken ovalbumin 44 kDa, equine myoglobin 17 kDa, and vitamin B_12_ 1.35 kDa). Yeast 20S and EcHslV were used as additional molecular mass standards.

### Analytical ultracentrifugation

AUC sedimentation velocity and sedimentation equilibrium experiments were performed using a Beckman-Coulter Optima XL-I (Indianapolis, USA) instrument equipped with AN-Ti 60 and AN-Ti 50 rotors, respectively. Absorbance scans were recorded at 280 nm. All experiments were performed at 20 °C in buffer D.

The sedimentation velocity assays were performed at 40,000 rpm using Epon double-sector cells, filled with 0.40 ml of protein and 0.41 ml of buffer D. Before the experiments, the samples were equilibrated in a centrifuge at 20 °C and 0 rpm for 1 h. Data were analyzed with the “Continuous c(s) distribution model” using the SEDFIT program [51]. The confidence level (*F*-ratio) was set to 0.68.

The sedimentation equilibrium experiments were performed using two-channel centerpieces, filled with 150 μl of the protein samples and 160 μl of buffers B and D in the reference sectors for YbAnbu and YbATP-grasp, respectively. The samples were equilibrated at 4 °C at a desired speed, and equilibrium was monitored for scans collected every 6 h, by “Test approach to equilibrium” procedure, from SEDFIT program, ver. 15.01c. Data were acquired every 0.001 cm with 20 replications using a step scan mode at speeds of 4000, 7000 and 9000 rpm. Data were analyzed using the multi-speed sedimentation equilibrium “Species analysis” procedure of the SEDPHAT program, ver. 13.0b [52].

The partial specific volume of the protein for all experiments was calculated with SEDNTERP program [53]. Density (*ρ* =1.00244 g/cm^3^ for buffer B and *ρ* =1.01603 g/cm^3^ for buffer D) and viscosity (*η* = 1.012 mPa s) of the buffer were measured using Anton Paar DMA 5000 (Graz, Austria) densitometer and Anton Paar Lovis 2000 M/ME viscosity-meter, respectively. The results were normalized and plotted using GUSSI program (Chad Brautigam, UT Southwestern).

### SAXS

SAXS data were collected at EMBL BioSAXS beamline X33 DESY, Hamburg, in the scattering length range of 0.067 nm as described earlier [54]. YbAnbu as well as controls EcHslV (smaller than Anbu) and Sc20S (bigger than Anbu) were used in a concentration of 1–5 mg/ml. The samples for the SAXS experiment were taken from the peak fractions of gel filtration on a Superose 6 column without any further concentration step. YbAnbu and EcHslV were in buffer D, while Sc20S proteasome was in buffer containing 50 mM Hepes (pH 7.4), 150 mM NaCl, and 1 mM NaN_3_.

### Electron microscopy

For negative-stain EM, YbAnbu sample (9 mg/ml) was initially mixed with tobacco mosaic virus in a 20:1 ratio. The protein mixture was then applied on a discharged copper grid covered with thin carbon layer for 30 s. The grid was washed four times with buffer B diluted 1:1 with 2% uranyl formate and dried. Data were collected on JEM-2100F operated at 200 kV and at calibrated 78,473-fold magnification. A total of 7981 particles were manually selected from micrographs using EMAN boxer [55], phase flipped using CTFFIND3 [56] and extracted to a stack by EMAN2 [57]. Reference-free 2D class averaging was performed using SPARX ISAC [58].

### Crystallization

Native crystals of YbAnbu grew by sitting drop vapor diffusion at 18 °C within 1–2 days. Five microliters of reservoir buffer containing 100 mM BIS–TRIS propane/HCl (pH 6.7–7.1), 200 mM Na_2_SO_4_, 18% polyethylene glycol 3350 and 7.5% ethylene glycol were mixed with 5 μl protein solution (concentration 8–20 mg/ml). Strong dependence between pH of the crystallization buffer and crystal quality was observed. In higher pH, crystals were bigger, were macroscopically multiple and grew in the presence of spherulites. In lower pH, crystals were smaller but of higher quality.

### Data collection and crystal structure determination

Diffraction data were collected at the PXI (X06SA) of the Swiss Light Source at 0.97988-Å wavelength. The data were processed with XDS and scaled with XSCALE [59]. Molecular replacement was performed with the PHASER software [26] and a monomer of HyAnbu (*P*2_1_2_1_2_1_ crystal form). The program found 13 Anbu subunits (TFZ between 4.9 and 17.7, LLG 2357). The model composed of two tetradecamers was manually assembled based on the dimerization and then tetramerization modes from the partial model and the reference structure. The resulting structure was then improved using the PHENIX [27] and ARP/wARP [28] rebuilding routines. The refinement was carried out with the help of PHENIX [27], REFMAC [60] and COOT [61] programs. The correctness of the final model was verified with MolProbity [62] and PDB validation tools.

### Structure analysis

The sequence conservation was analyzed with the Consurf program (PsiBlast alignment with 10^−4^ E-value cutoff and maximum 500 sequences, but other options led to very similar results). The structural similarity to other proteins was analyzed using the PDBeFold server [29].

## Supplementary Material

Supp

## Figures and Tables

**Table 1.** Data collection and refinement statistics

Space group	*P*2_1_
Unit cell dimensions	
*a, b, c* (Å), *β* (°)	95.5, 285.4, 179.2, 91.8
Resolution (Å)	50–2.5
Lowest shell	50–22.5
Highest shell	2.55–2.50
Total reflections	1,883,902
Unique reflections	325,849
Completeness (%)	98.9 (90.3, 95.1)
Multiplicity	5.8 (5.6, 5.7)
*∣*/σ*∣*	14.6 (50.0, 2.1)
*R*_sym_	8.7 (3.2, 97.5)
*R*_merge_	9.2 (3.0, 107.8)
Solvent content (%)	63.4
B(iso) from Wilson (Å^2^)	61.1
*R*_work_/*R*_free_ (%)	18.28/22.98
No. atoms	52,945
Protein	51,008
Ligand/ion	147
Water	1790
Average *B*-factor (Å^2^)	65.0
R.m.s deviations	
Bond lengths (Å)	0.009
Bond angles (°)	1.2

Values for the lowest- and highest-resolution shell are given in parentheses. Numbers of atoms correspond to the multiple conformers counted separately.

## Abbreviations used:

HslV: heat shock locus V
Anbu: ancestral beta subunit
EM: electron microscopy
SAXS: small-angle X-ray scattering
