# Supplementary material for: The Y. bercovieri Anbu crystal structure sheds light on the evolution of highly (pseudo)symmetric multimers
Source: J Mol Biol. Author manuscript; Available in PMC 2019 Feb 15. (PMC6376114; doi:10.1016/j.jmb.2017.11.016)
Supplement: Supp [file NIHMS1010391-supplement-Supp.pdf]

Supplementary Material for:

The *Y. bercovieri* Anbu crystal structure sheds light on the evolution of highly (pseudo)symmetric multimers

Anna Piasecka[1,2], Honorata Czapinska[1,3], Marie-Theres Vielberg[4], Roman Szczepanowski[3], Reiner Kiefersauer[5], Simon Reed[2], Michael Groll[4], Matthias Bochtler[1,3,6,#]

[1]*Polish Academy of Sciences, Institute of Biochemistry and Biophysics, Warsaw, Poland*

[2]*Cardiff University, School of Medicine, Cardiff University, Cardiff, United Kingdom*

[3]*International Institute of Molecular and Cell Biology, Warsaw, Poland*

[4]*Center for Integrated Protein Science at the Department Chemie, Lehrstuhl für Biochemie, Technische Universität München, Garching, Germany*

[5]*Proteros biostructures GmbH, Martinsried, Germany*

[6]*Cardiff University, Schools of Chemistry and Biosciences, Cardiff, United Kingdom*

[#]Corresponding author: mbochtler@iimcb.gov.pl

Suppl. Fig. 1:

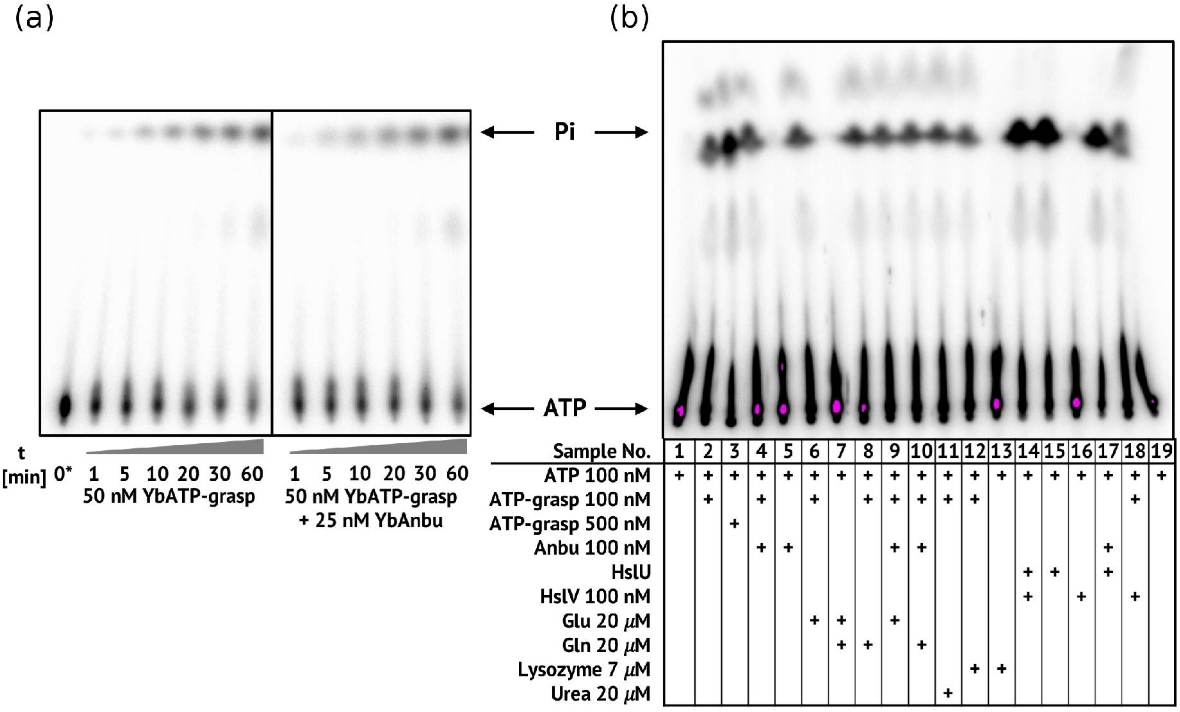

Sample No.	1	2	3	4	5	6	7	8	9	10	11	12	13	14	15	16	17	18	19
ATP 100 nM	+	+	+	+	+	+	+	+	+	+	+	+	+	+	+	+	+	+	+
ATP-grasp 100 nM		+		+		+		+	+	+	+	+						+	
ATP-grasp 500 nM			+																
Anbu 100 nM				+	+				+	+							+		
HslU														+	+		+		
HslV 100 nM														+		+		+	
Glu 20 μM						+	+		+										
Gln 20 μM							+	+		+									
Lysozyme 7 μM												+	+						
Urea 20 μM											+								

Suppl. Fig. 1: (A) Time course of YbATP-grasp catalyzed orthophosphate release from γ-P^{32}-ATP in the absence (top) and presence (bottom) of YbAnbu. 0* designates the full incubation time (60min) in the absence of an enzyme. This panel reports the same experiment as Fig. 1E, but the full autoradiogram covering the entire TLC plate is shown. **(B)** Orthophosphate release by the YbATP-grasp protein in the presence of the indicated additional components (lysozyme was tested as an unrelated control protein).

Suppl. Fig. 2:

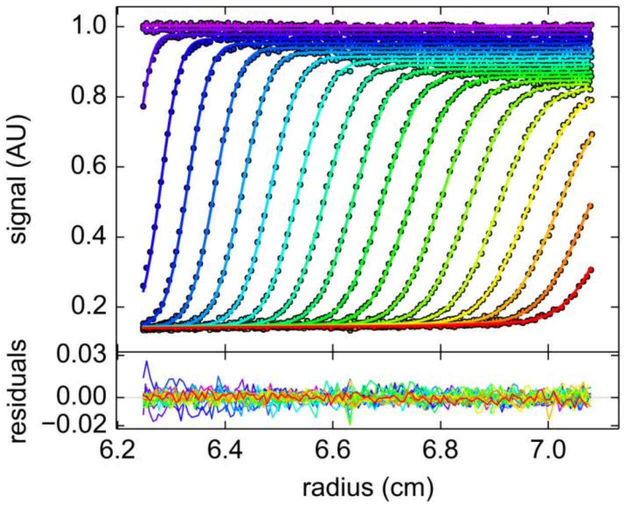

Suppl. Fig. 2: Sedimentation velocity analysis of Anbu scanned by absorbance at 280 nm. Sedimentation coefficient distribution profiles indicated average sedimentation coefficient of 12.6 S ($sw_{(20,w)}$=12.8 S). A corresponding weight average molecular weight ~ 342 kDa, based on v-bar= 0.731 ml g^{-1}, buffer density = 1.00012 g/cm^3 and buffer viscosity 1.012 mPa s. Every third scan and every third point is shown for clarity.

Suppl. Fig. 3:

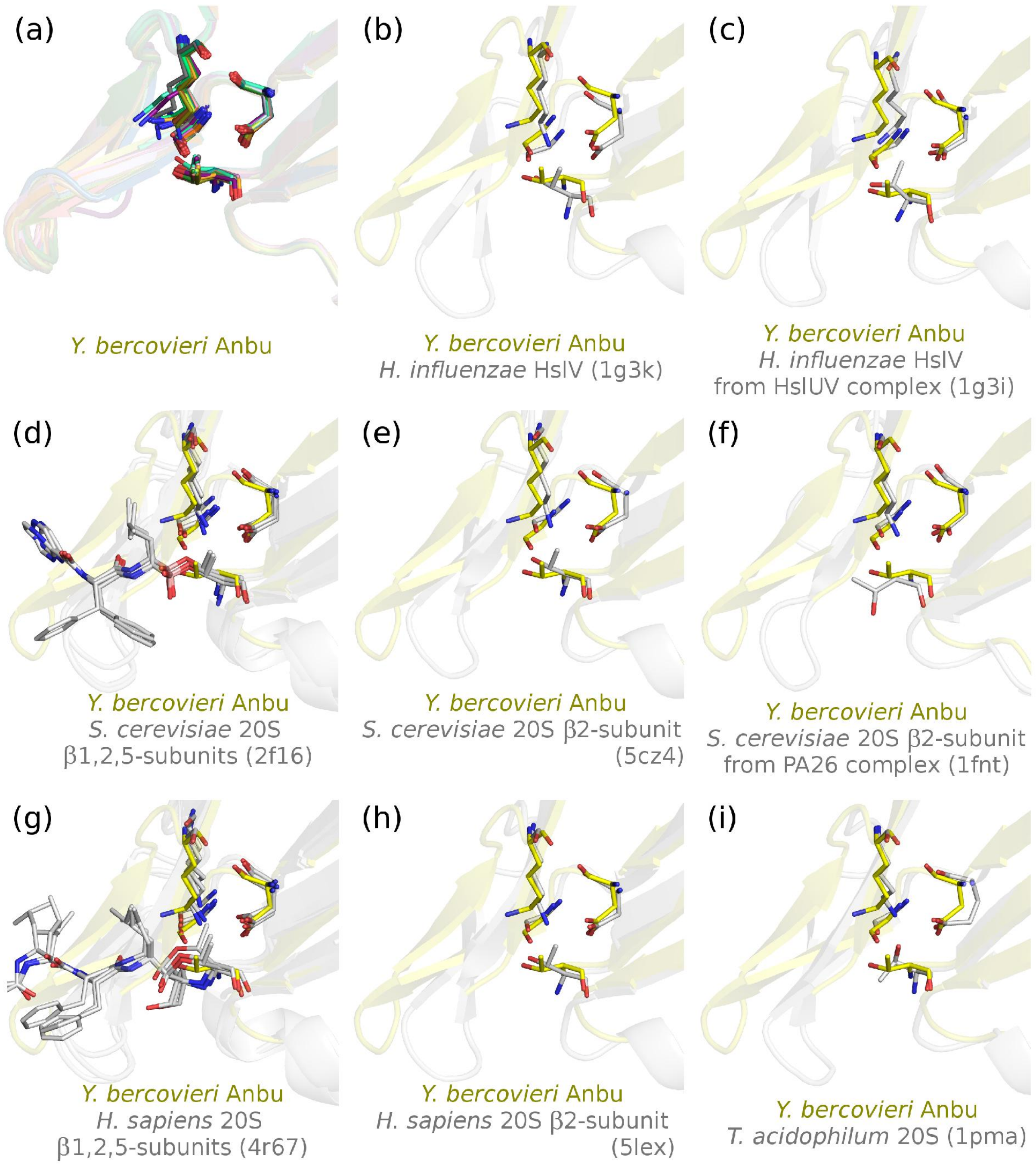

Suppl. Fig. 3: Gallery of 20S and HslV proteasome active sites (gray) overlaid with YbAnbu (yellow). The key catalytic residues are shown in stick representation. The side chain of R19 is omitted for clearance. The superposition of: **(A)** the putative active site residues in all 28 subunits of the two tetradecameric particles of YbAnbu in the asymmetric unit. **(B,C)** YbAnbu and HslV active site in either **(B)** free HslV or **(C)** HslV in complex with its HslU activator; **(D-F)** YbAnbu and Sc20S in **(D)** bortezomib bound form (all three catalytic subunits are overlaid), **(E)** inhibitor free form or **(F)** *Trypanosome brucei* PA26 activator bound form; **(G-H)** YbAnbu and Hs20S in **(G)** carfilzomib bound form (all three catalytic subunits are overlaid) or **(H)** inhibitor free form; and **(I)** YbAnbu and Ta20S proteasome. Notice the different conformations of the β-hairpin in YbAnbu and in proteasomes.

Suppl. Fig. 4:

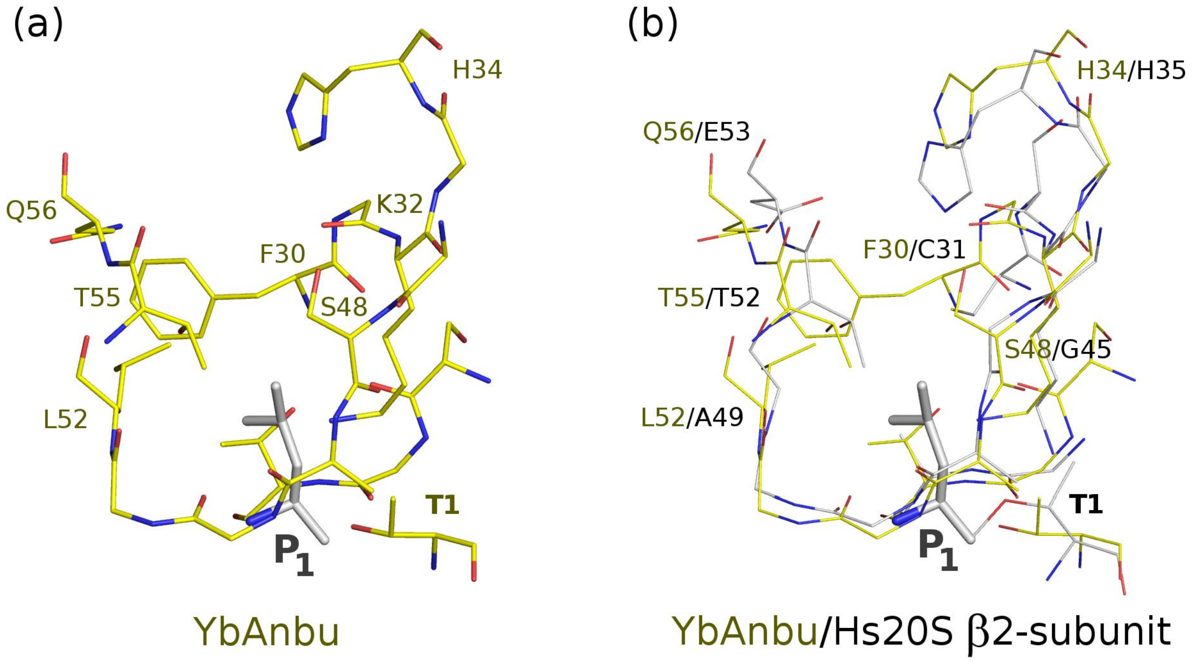

Suppl. Fig. 4: The YbAnbu S_1 pocket resembles the proteasomal β2-subunit pocket, but is narrower. (A) Putative S_1 pocket of YbAnbu with P_1 residue mapped from the co-crystal structure of Hs20S with carfilzomib (PDB 4r67 [61]) based on global superposition of subunits. Key residues are shown in stick representation. Some side chains have been omitted for clearance. **(B)** Superposition of YbAnbu (yellow) and Hs20S β2 (gray) to illustrate the similar location of the key residues. The figure is 90° rotated with respect to Fig. 5 in the article.

Suppl. Fig. 5:

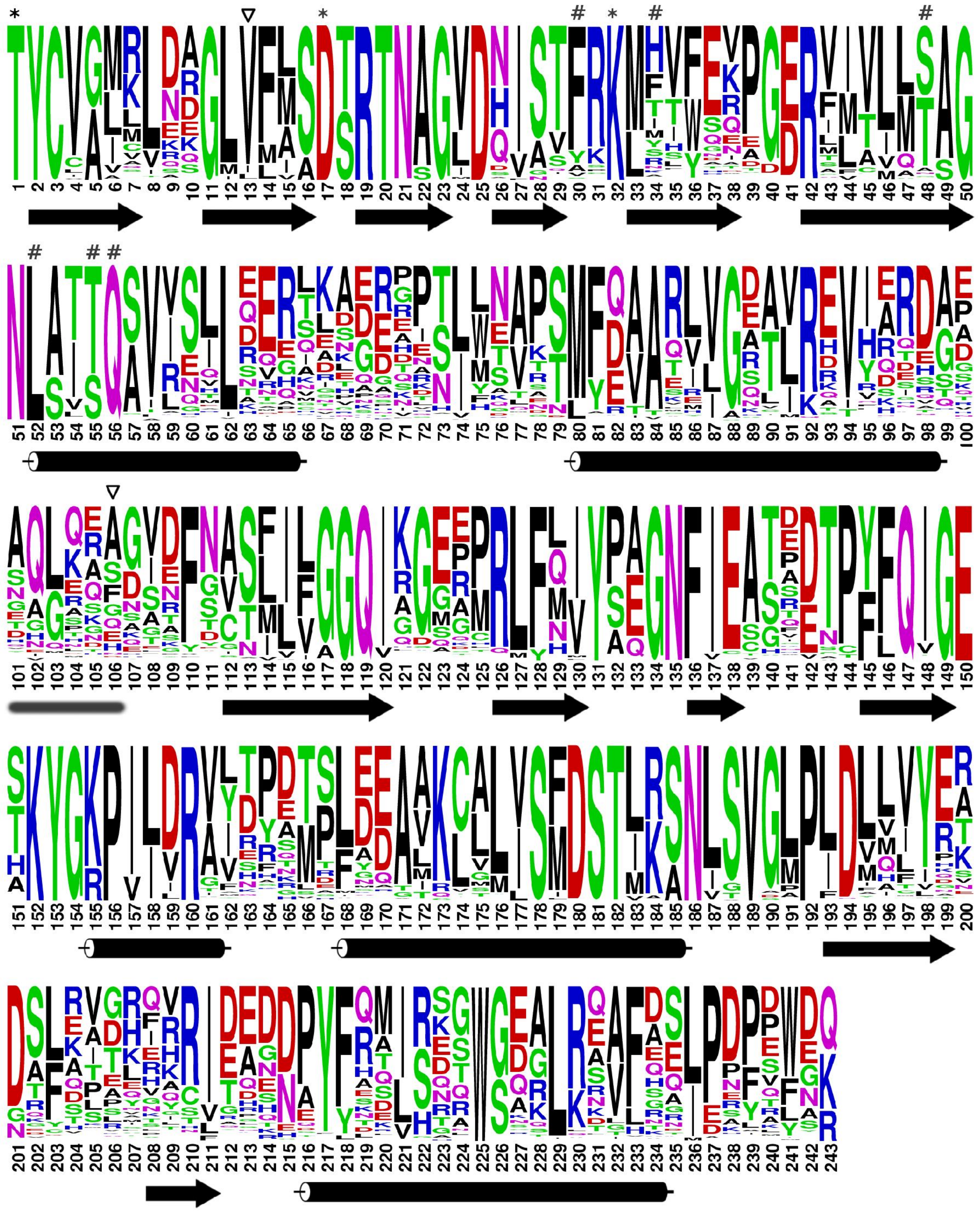

Suppl. Fig. 5: Anbu sequence logo in frequency representation generated automatically by Consurf using PSI-BLAST aiming for at most 500 sequences [63]. Numbering and annotation of secondary structure refer to YbAnbu (omitting sequence positions absent in YbAnbu). Secondary structure was analyzed using PROMOTIF [64] and refers to YbAnbu. The blurred bar in the secondary structure annotation indicates the disordered region in the YbAnbu structure. The * symbols mark residues corresponding to active site residues in HslV and 20S proteasomes. The # symbols indicate key residues of the putative S_1 pocket. The ∇ symbols designate residues that have been deliberately or accidentally altered with respect to the YbAnbu sequence in the UNIPROT database (entry A0A0T9RXH3, gene ERS008506_03702).

Suppl. Fig. 6:

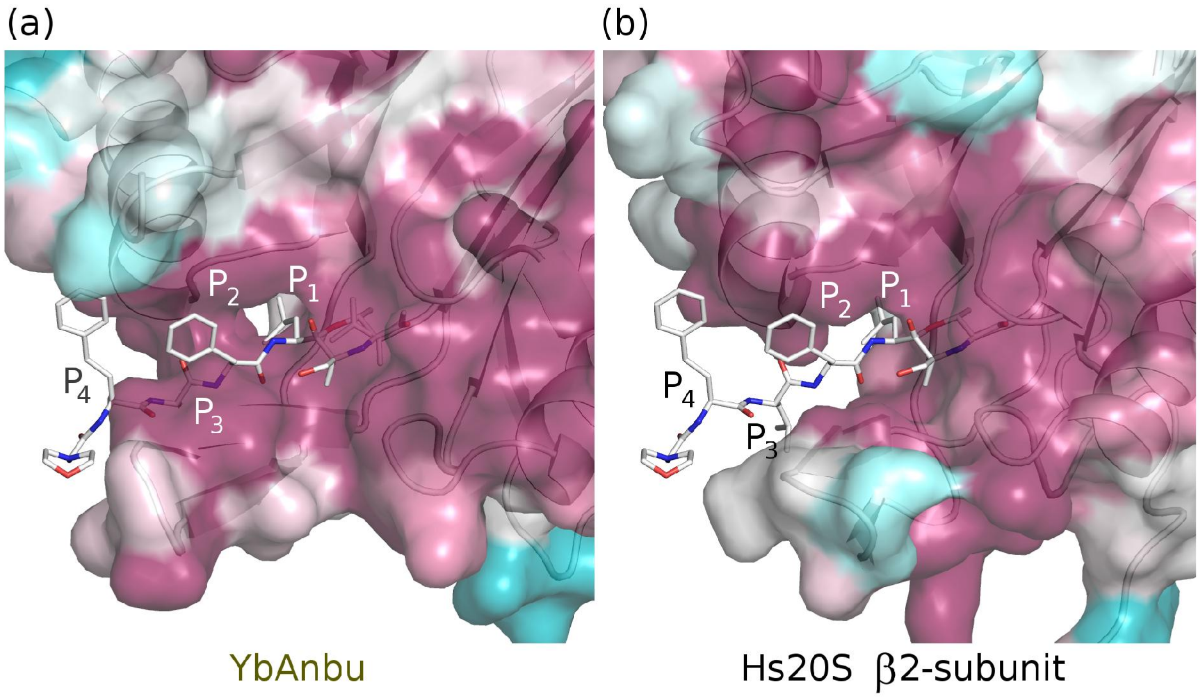

Suppl. Fig. 6: The putative substrate binding site of YbAnbu is occluded by a β-haripin. (A) The the putative substrate binding site of Anbu. **(B)** The binding site of the β2 subunit of human 20S proteasome in complex with carfilzomib (PDB 4R67). Carfilzomib in (A) has been modelled based on its conformation in the Hs20S structure shown in (B).

Suppl. Fig. 7:

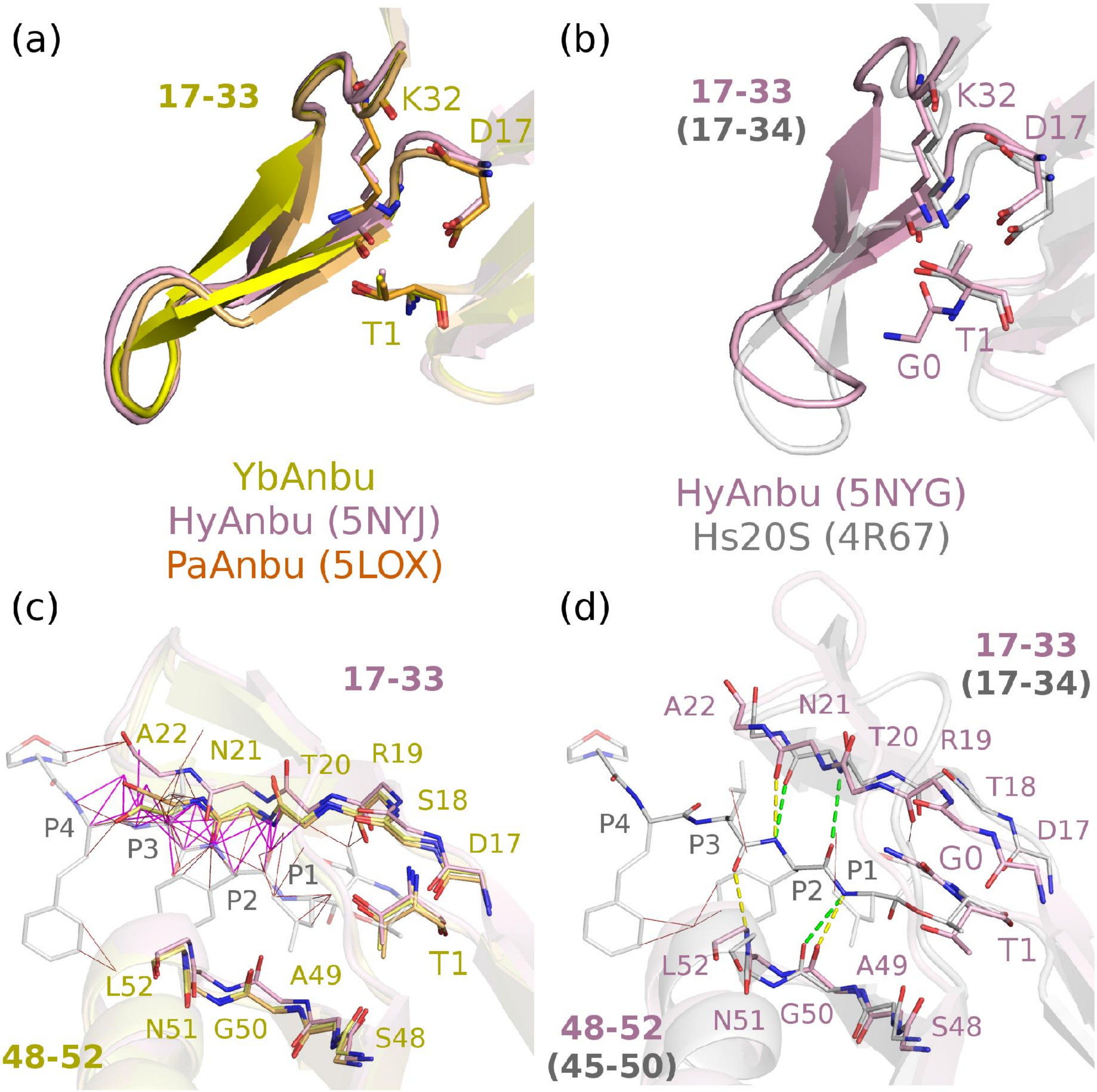

Suppl. Fig. 7: Comparison of the active site regions in published Anbu structures and human 20S proteasome. (A) The 17-33 loop conformation in the YbAnbu structure as well as in HyAnbu crystallized in *C*2 space group (PDB 5NYJ) and PaAnbu (PDB 5LOX) is markedly different from the one observed in **(B)** Hs20S proteasome (PDB 4R67, gray) and HyAnbu G0 variant (PDB 5NYG), which adopts the form known for 20S and HslV proteasomes. **(C)** The loop 17-33 loop in the unusual position observed for some Anbu structures would severely clash with the substrate/inhibitor as depicted by the model of the complex based on the Hs20S-carfilzomib structure. The main chain – main chain clashes are indicated by thick magenta lines, all other clashes by thin red lines. **(D)** For the 20S-like loop position observed for the G0 HyAnbu variant, the main chain – main chain clashes are missing and only very limited number of other clashes is observed (thin red lines). Instead, the hydrogen bonds observed in the Hs20S structure (green) can potentially be formed (yellow).

Suppl. Fig. 8:

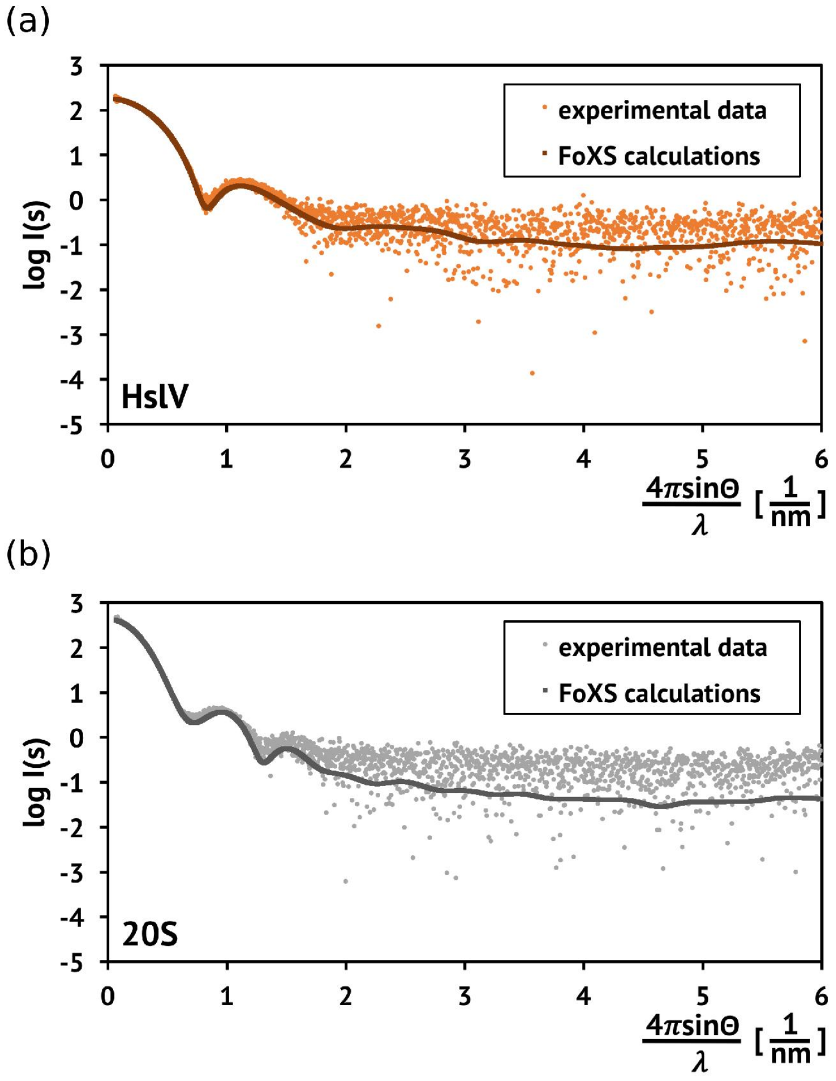

Suppl. Fig. 8: Fitting of SAXS data that were experimentally measured (dots) to the data calculated based on the crystal structures (curve). The χ parameter, a measure for the quality of the fit, is **(A)** χ=1.16 for EcHslV and **(B)** χ=1.61 for Sc20S.

Suppl. Fig. 9:

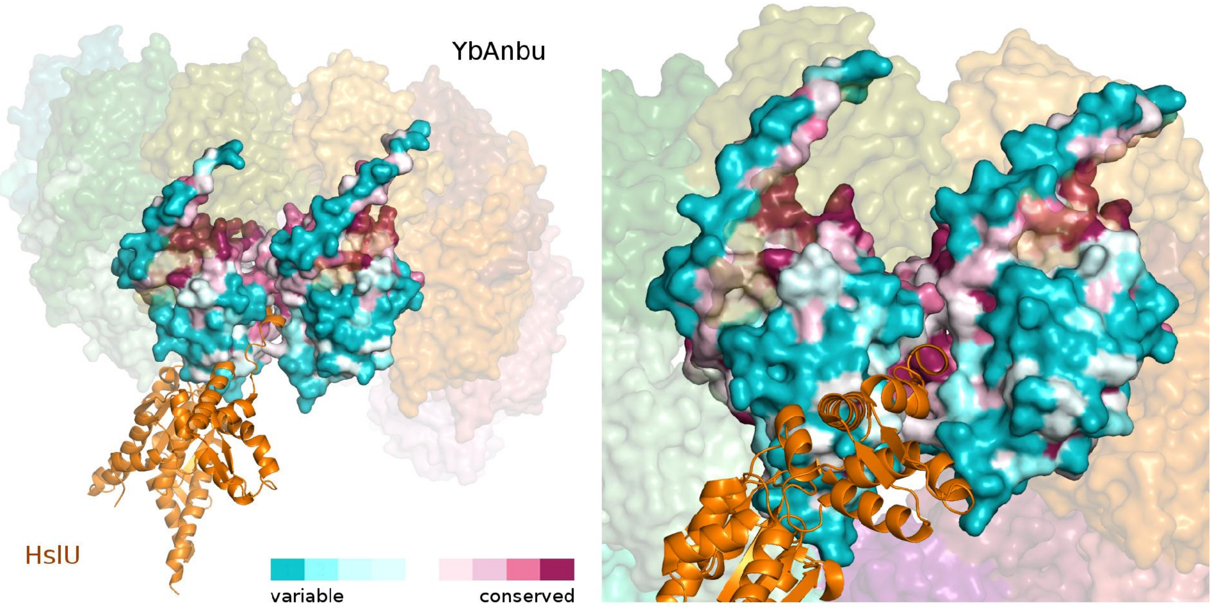

Suppl. Fig. 9: Superposition of HslUV on YbAnbu to demonstrate that YbAnbu shares with HslV the groove that accommodates the C-terminal tail of HslU. However, we also note that YbAnbu activity remains elusive and that there is no experimental evidence for HslU or YbATP-grasp stimulation of Anbu.

Suppl. Fig. 10:

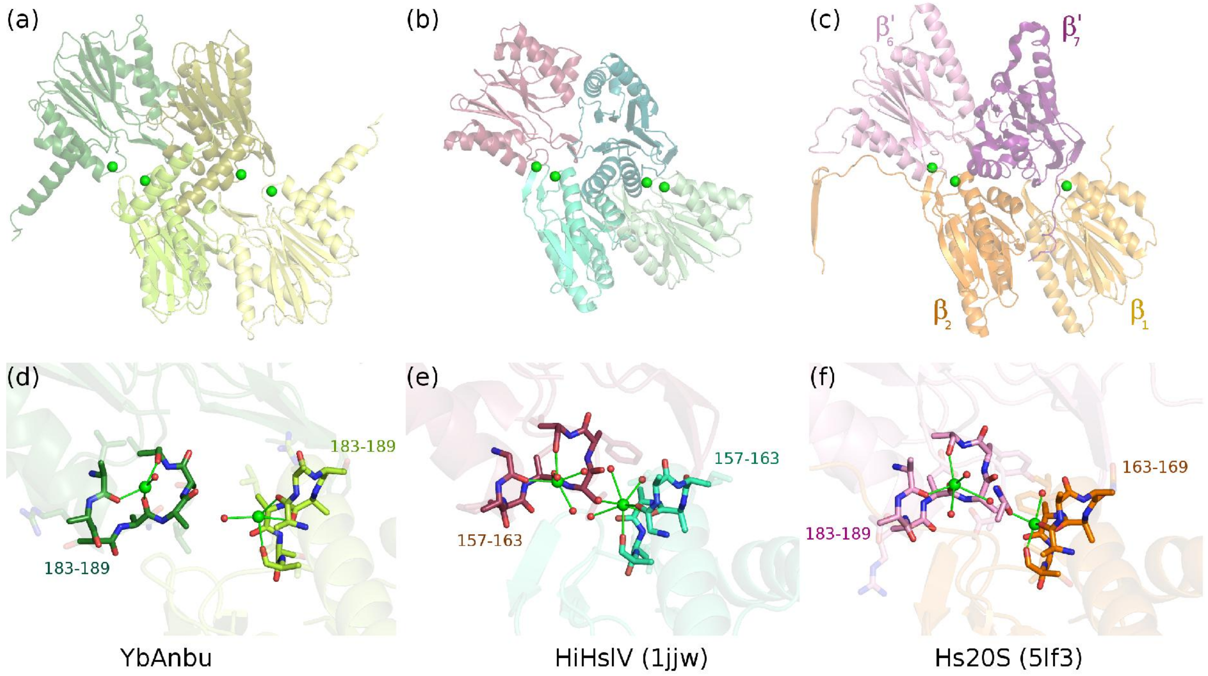

Suppl. Fig. 10: Conserved cation binding sites in the structures of YbAnbu, HslV and 20S proteasomes. Only two protomers (four monomers) from the **(A)** YbAnbu, **(B)** HiHslV and **(C)** 20S proteasome are shown in a view from the outside of the ring or lock washer. The green balls indicate the conserved binding sites most likely occupied by waters in the Anbu structure, K^+ ions in the HslV structure and either K^+ or Mg^{2+} ions in the 20S structure. **(D-E)** Enlarged view of the ion binding sites indicating the conserved positions of the main chain carbonyl groups coordinating the ions (side chains are indicated in a faint mode to make the picture clearer).

Suppl. Fig. 11:

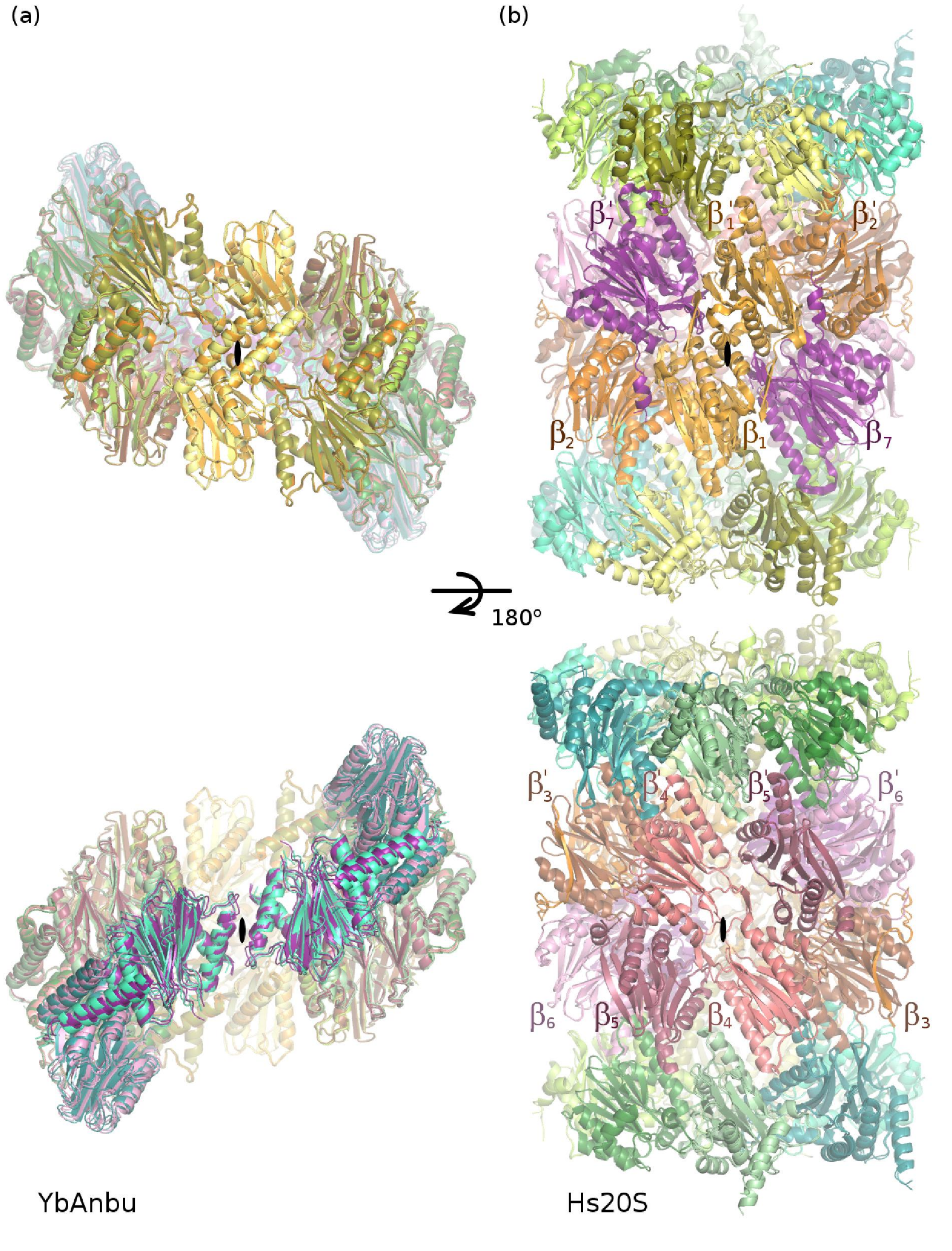

Suppl. Fig. 11: Two-fold symmetry of YbAnbu and (B) 20S proteomes. The two-fold symmetry axis runs perpendicularly to the page (indicated by an oval). **(A)** Two YbAnbu particles present in the asymmetric unit of the crystal have been overlaid and then mapped on top of themselves according to the internal two fold symmetry axes of the molecules. **(B)** The 20S proteasome particle (PDB: 5FL3) has been mapped according to the two fold axis of the molecule.
